# Unveiling the Potential of Surface Polymerized Drug Nanocrystals in Targeted Delivery

**DOI:** 10.1021/acsami.4c07669

**Published:** 2024-08-28

**Authors:** Jakes Udabe, Sergio Martin-Saldaña, Yushi Tao, Matías Picchio, Ana Beloqui, Alejandro J. Paredes, Marcelo Calderón

**Affiliations:** † POLYMAT, Applied Chemistry Department, Faculty of Chemistry, University of the Basque Country UPV/EHU, Paseo Manuel de Lardizabal 3, 20018 Donostia-San Sebastián, Spain; ‡ School of Pharmacy, Queen’s University Belfast, 97 Lisburn Road, Belfast, Northern Ireland BT9 7BL, U.K.; § IKERBASQUE, Basque Foundation for Science, Plaza Euskadi 5, 48009 Bilbao, Spain

**Keywords:** nanocrystals, polyethyleneglycol (PEG), mucin, surface chemistry, nose-to-brain route, targeted delivery

## Abstract

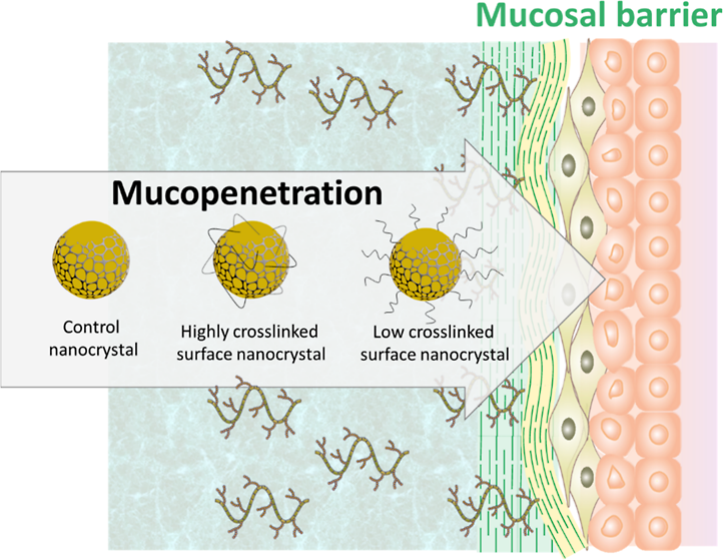

Nanocrystals (NCs) have entirely changed the panorama of hydrophobic drug delivery, showing improved biopharmaceutical performance through multiple administration routes. NCs are potential highly loaded nanovectors due to their pure drug composition, standing out from conventional polymers and lipid nanoparticles that have limited drug-loading capacity. However, research in this area is limited. This study introduces the concept of surface modification of drug NCs through single-layer poly(ethylene glycol) (PEG) polymerization as an innovative strategy to boost targeting efficiency. The postpolymerization analysis revealed size and composition alterations, indicating successful surface engineering of NCs of the model drug curcumin of approximately 200 nm. Interestingly, mucosal tissue penetration analysis showed enhanced entry for fully coated and low cross-linked (LCS) PEG NCs, with an increase of 15 μg/cm^2^ compared to the control NCs. In addition, we found that polymer chemistry variations on the NCs’ surface notably impacted mucin binding, with those armored with LCS PEG showing the most significant reduction in interaction with this glycoprotein. We validated this strategy in an in vitro nose-to-brain model, with all of the NCs exhibiting a promising ability to cross a tight monolayer. Furthermore, the metabolic and pro-inflammatory activity revealed clear indications that, despite surface modifications, the efficacy of curcumin remains unaffected. These findings highlight the potential of surface PEGylated NCs in targeted drug delivery. Altogether, this work sets the baseline for further exploration and optimization of surface polymerized NCs for enhanced drug delivery applications, promising more efficient treatments for specific disorders and conditions requiring active targeting.

## 1 Introduction

Improving drug absorption is a big hurdle for the pharmaceutical industry, as 70% of drugs have poor bioavailability.^1^ In this context, drug nanocrystals (NCs) represent an exciting class of nanomaterials that can deliver poorly soluble drugs due to their unique size-dependent properties, playing a pivotal role in various clinical applications.^2,3^ Their small dimensions, typically from 100 to 500 nm, lead to a high surface area-to-volume ratio, contributing to their exceptionally increased saturation solubility and dissolution rate.^4^ Consequently, more dissolved drug molecules become available for absorption across physiological barriers, facilitating subsequent systemic distribution, pharmacokinetics, and bioavailability.^5^ These distinctive attributes render NCs particularly advantageous for class II drugs, typified by low solubility and high permeability, positioning them as a widely embraced platform for boosting the in vivo performance of poorly soluble drugs.^6^ Furthermore, NCs offer advantages over drugs loaded in traditional nanocarriers, such as polymeric or lipid-based nanoparticles, regarding drug-loading capabilities as they have a minimal amount of carrier materials, offering a full drug-loading capacity.^5^ Their ability to finely tune dimensions and their potential for reducing side effects by decreasing doses, controlling drug release, and enhancing therapeutic efficacy make NCs particularly promising in ocular, pulmonary, transdermal, and intranasal drug delivery.^7^ Understanding the synthesis, properties, and applications of these nanoscale materials is essential for advancing fundamental research and practical technological innovations.^8^

Despite efforts to design NCs for rapid dissolution, they often remain intact for a given timespan, influenced by factors such as route administration and drug characteristics.^9^ Such lag time before the dissolution of the NCs creates an interesting opportunity to use intact NCs as highly loaded nanovectors to target tissues and cells.^10^

Unfortunately, despite their advantageous capacities, NCs present significant limitations in target selectivity.^11^ Unlike polymeric nanoparticles with the potential for surface modifications and functionalization,^12,13^ targeted drug delivery with NCs is challenging due to a lack of precision, reducing their efficiency and therapeutic outcome. Further, most approaches to modifying NC surface do not guarantee the preservation of the pharmacokinetic activity of the active compound, as these modifications may potentially alter its chemical structure.^14^ Interfacial cohesion and supramolecular assembly with metal-phenolic networks have recently been proposed for NCs’ surface engineering, although the stability of the metal–ligand coordination at different pH levels is a main pitfall for this strategy.^15^ Hence, surface customization emerges as a promising strategy to enable targeted delivery to specific cells and tissues before dissolution, presenting an intriguing avenue for investigation.

Notably, there is still a lack of successful surface engineering approaches that effectively allow active targeting while suppressing Ostwald ripening phenomena,^4^ meaning preserving the integrity of the NCs. In particular, in the context of neurological treatments, the challenges in achieving effective drug delivery are closely linked to the need for selectivity due to the high complexity of the central nervous system (CNS) and the barriers protecting it. Hence, the most simplistic administration approach is the so-called nose-to-brain route,^16^ offering a direct path through the olfactory and trigeminal nerves while minimizing side effects related to drug systemic circulation. Nonetheless, precision in targeting is crucial when drugs are transported from the nasal cavity to the brain. Without selectivity, drug delivery efficiency to the brain is significantly reduced.^16^ Selectivity is critical in ensuring that the drug reaches the intended brain regions or specific cells, which is particularly challenging when dealing with NCs due to their inherent lack of surface specificity.

Polymer surface chemistry could play a pivotal role in addressing the challenges related to NCs. Tailoring the surface of NCs through polymer chemistry techniques is an entirely unexplored concept that would enable the introduction of specific functional groups, coatings, or modifications for enhanced selectivity. This engineered surface approach will allow precise targeting by facilitating interactions with specific cells, tissues, or organs, influencing drug release kinetics, and improving NC stability.

**Scheme 1:**
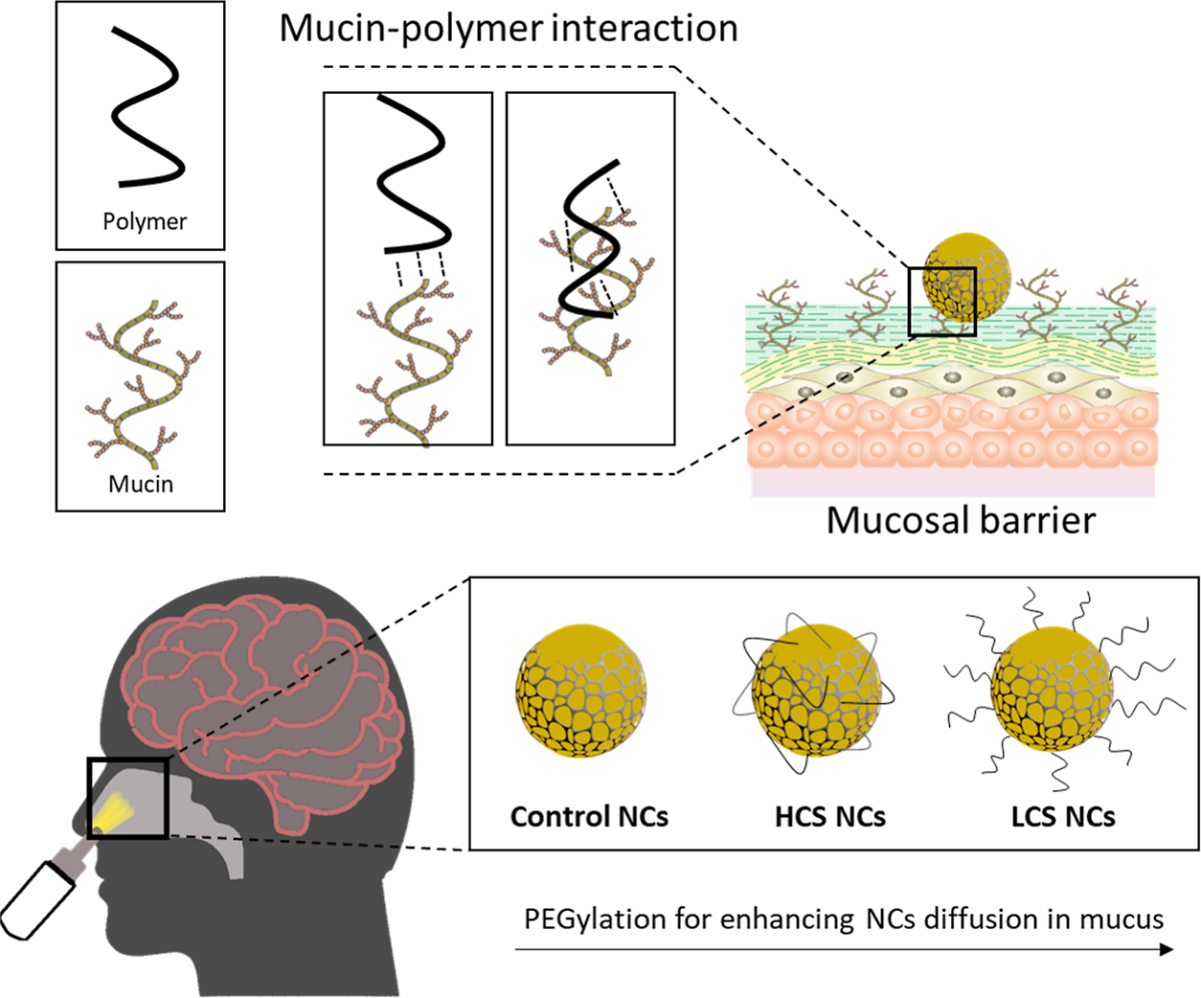
Schematic Representation of the Mucin-Polymer Interaction and How It Could Enhance NC Diffusion in Mucus by PEGylation, the Control NCs Refer to Polymerized NCs from Reactive Poloxamer, HCS NCs Were Obtained Using PEG-Diacrylate, While LCS NCs Were Obtained Using PEG-Acrylate

Herein, we propose for the first time surface-initiated polymerization as an innovative tool for NC engineering. Different surface PEGylation polymerization strategies were performed to test the role of surface nanoarchitectures on NCs’ interaction with biological barriers. To demonstrate our concept, we work out a nose-to-brain drug delivery model engineering NCs for mucin binding. For that, the NCs were stabilized using various formulations of mucopenetrating poloxamers^17^ and subsequently surface-modified with polyethylene glycol (PEG) units. PEG surface chemistry was explored since this polymer is well-known for reducing mucin interaction, as previously reported for polymeric or lipid nanoparticles.^18,19^ Mucin typically impedes the efficient transport of drugs to the brain, and PEG-decorated NCs could potentially decrease their affinity with this glycoprotein, thus enhancing their capability to traverse the mucosal barrier for more effective nose-to-brain drug delivery.^20,21^ Hence, we systematically investigated the effect of surface chemistry and topology on mucin binding by using various PEG-based polymerizable motifs to create high cross-linking surface (HCS) and low cross-linking surface (LCS) polymer layers around the NCs. These were compared with different ratios of PEGylation, self-PEGylation, and non-PEGylated NCs to determine the effect of this surface modification. This approach aimed to modulate their stability and drug release kinetics. Additionally, we compared these systems with a control stabilized by a mucopenetrating PEG-containing surfmer (polymerizable poloxamer) (Scheme 1).

## 2 Materials and Methodology

### 2.1 Materials

Ammonium persulfate (APS), curcumin (CUR) (extracted from curcuma longa (turmeric), CAS: 458-37-7), mucin from the porcine stomach (Type II), poly(ethylene glycol)_100_-*block*-poly(propylene glycol)_65_-*block*-poly(ethylene glycol)_100_ (PEG-PPG-PEG, poloxamer) (MW 12.5 kDa), PEG-PPG-PEG-diacrylate (MW 12.5 kDa) (poloxamer diacrylate), PEG-acrylate (MW 2 kDa), and PEG-diacrylate (MW 2 kDa) were purchased from Sigma-Aldrich (Spain). Methanol (MeOH) (>99.9%), Tween 80, and tetramethylethylenediamine (TEMED) were obtained from Sigma-Aldrich (Dorset, UK). Ceramic beads of 0.1 to 0.2 mm were purchased from Chemco International (Guangfu, China). Magnetic stirring bars (25 × 8 mm) and dialysis membrane with MW cutoff (MWCO) of 14 kDa were purchased from Scharlau (Spain). Phosphate buffered saline (PBS) pH 7.4 tablets were purchased from Oxoid Limited (Hampshire, UK). High-performance liquid chromatography water was obtained from a water purification system, Elga Purelab DV 25, Veolia Water Systems (Ireland). All other chemicals used were of analytical grade.

### 2.2 Preparation of CUR NCs

The size of the NCs was chosen to be approximately 200 nm, as this hydrodynamic size is considered optimal for developing mucopenetrating nanoparticles, according to the literature.^22^

A recent study of an orthogonal approach for CUR NC particle size and polydispersity design revealed that various approaches, such as stabilizers, time, and fluid dynamics, directly affect particle size and polydispersity.^23^ Consequently, after a preliminary analysis considering different polymer concentrations and milling times, along with a previously reported media milling technique,^9,24^ robust NCs with a size of 200 nm and relatively low polydispersity were successfully designed, resulting in NCs with favorable mucopenetrating properties.

**Scheme 2:**
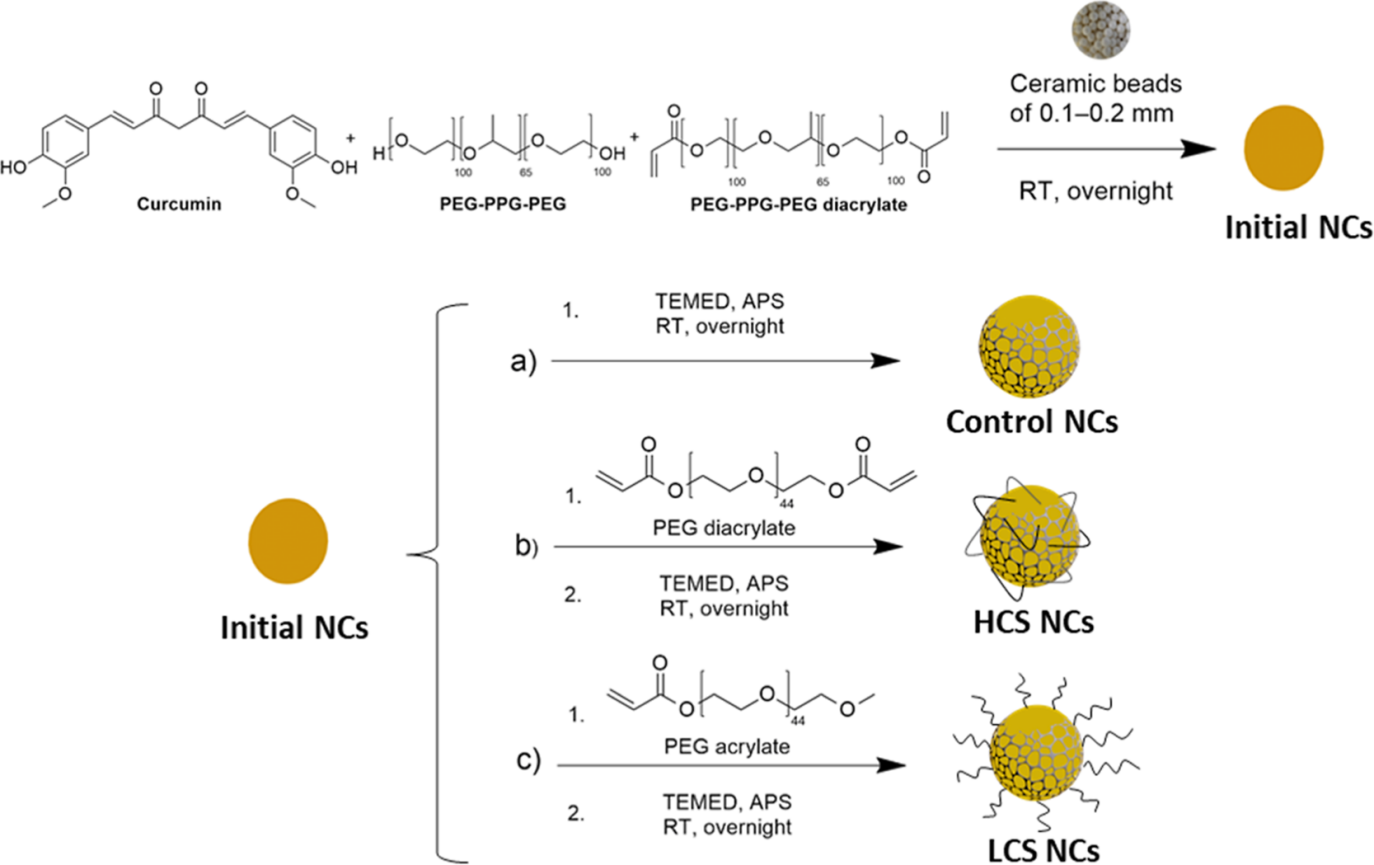
Synthesis of Initial NCs and Post-Polymerization to Prepare (A) Control NCs, (B) HCS NCs, and (C) LCS NCs

CUR NCs were prepared by dispersing 100 mg of CUR in 5 mL of 0.5 wt % polymer surfactant solution in a 10 mL glass vial with 1.5 mL of ceramic beads as the milling media, followed by two magnetic stirring bars (25 × 8 mm). The system was hermetically sealed and wrapped with aluminum foil to protect the drug from light. After that, it was placed on an IKA RCT Basic Magnetic Stirrer (Staufen, Germany) at a fixed rotation speed of 1200 rpm for 24 h of milling; the CUR NCs were separated from the beads and magnets using a filter (300-mesh sieve, 74 μm-pore size). Five formulations were prepared by variating the polymer surfactant composition using a nonreactive and a reactive stabilizer in different wt % ratios (PEG-PPG-PEG: PEG-PPG-PEG-diacrylate, 100_0, 75_25, 50_50, 25_75, 0_100). An illustrative representation of the experimental setup is presented in Scheme 2. All dispersions were purified against deionized water using a dialysis membrane with a 14 kDa MWCO over 24 h in 1 L, changing the water three times, and the size and polydispersity index (PDI) evolution were measured by dynamic light scattering (DLS).

### 2.3 Surface Polymerization of CUR NCs

The surface of the NCs was modified by different polymerization processes. For control NCs, CUR NCs obtained from the milling process were dispersed in water at a final concentration of 1 mg/mL, and APS (1 mg) and TEMED (1 μL) were added and left overnight at room temperature (RT) stirring at 350 rpm. HCS NCs and LCS NCs were prepared following the same protocol but adding PEG-diacrylate or PEG-acrylate (1 mol equiv over vinyl groups), respectively, in the initial solution. Finally, the polymerized NCs were purified against deionized water for 48 h by using a dialysis membrane (14 kDa MWCO). The surface polymerization of the CUR NCs process is summarized in Scheme 2.

### 2.4 Physiochemical Characterization of NCs

#### 2.4.1 Particle Size, Size Distribution, and Zeta Potential

DLS was used to obtain the average hydrodynamic diameters and the PDI values of the NCs. A total of 1 mL of the NCs dispersion (1 mg/mL) was added to a polystyrene clear-sided cuvette when dispersed in Mili-Q water. The sample in the cuvette was well mixed, and then the size was analyzed by intensity on a Malvern Zetasizer Nano (Worcestershire, United Kingdom) at an angle of 173° and in the 4.65 mm height of the plastic cuvette. The same instrument was used to determine the surface zeta potential of the NCs. A total of 1 mL of the NC dispersion (1 mg/mL) in 10 mM NaCl was added to disposable folded capillary cells (Malvern Zetasizer) and measured at RT. All measurements were conducted in triplicate, and the data were presented as the mean ± standard deviation (SD).

#### 2.4.2 Composition Analysis

The chemical composition of the NCs was determined using Fourier transform infrared spectroscopy (FTIR) (FT/IR-4100 Series, Jasco, Essex, UK) in the wavelength range of 4000 to 500 cm^–1^, proton-1 nuclear magnetic resonance (^1^H NMR) (Bruker AVANCE 300), and carbon-13 NMR (^13^C NMR) (Bruker AVANCE 300), dissolving the NCs in DMSO-*d*_9_.

#### 2.4.3 Differential Scanning Calorimetry

Differential scanning calorimetry (DSC) was performed by using a Q100 differential scanning calorimeter system (TA Instruments, New Castle, DE, USA). The samples (5–10 mg) were placed into standard aluminum pans, sealed with a DSC lid, and heated in a nitrogen atmosphere at a stepping rate of 10 °C/min over a temperature range of 25–200 °C with an empty pan as a reference.

#### 2.4.4 X-ray Diffraction

X-ray diffraction (XRD) analysis was conducted using Bruker D8 Advance equipment. Approximately 20 mg of sample material was evenly spread onto a flat sample holder. The analysis was carried out in a controlled environment. The sample was subjected to X-ray irradiation at a constant angle, 2θ, while the detector recorded the diffraction pattern produced by the interaction of X-rays with the sample’s crystalline structure. Data collection was performed over a specified angular range, from 5 to 80° (2θ).

#### 2.4.5 Transmission Electron Microscopy

Transmission electron microscopy (TEM) analysis was conducted using a TECNAI G2 20 TWIN instrument (Donostia, Spain) operated at 200 kV and equipped with a LaB6 filament. For sample preparation, dispersion into water was carried out, and a drop of the suspension was applied to a TEM copper grid (300 mesh) covered with a pure carbon film. The grid was then air-dried at RT. Before the addition of the suspension drop, the grid underwent glow discharge treatment. The NC suspensions were diluted to 0.025 mg/mL with Milli-Q water and deposited on a TEM grid for microscopical analysis.

### 2.5 Drug Content Evaluation

The drug content of the NCs was determined by the absorbance measurement according to eq 1. Known amounts of NCs were dissolved in pure methanol, vortexed, and sonicated for 5 min. The supernatant was measured on a plate reader at a 425 nm wavelength and quantified using a calibration curve made with raw CUR dissolved in pure methanol (LOD = 0.5 μg/mL; LOQ = 1.5 μg/mL). All measurements were conducted in triplicate, and the data were presented as the mean ± SD
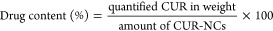
1

### 2.6 In Vitro Release Study

A dialysis membrane method was used to assess the in vitro release kinetics by comparing pure CUR with different NCs. Two media were used: (1) phosphate buffer with pH 7.4 at 10 mM concentration containing 1% w/v Tween 80 and (2) phosphate buffer with pH 7.4 with 10 mM concentration containing 1% w/v Tween 80 and 1% w/v ascorbic acid to prevent CUR oxidation. To ensure the sink conditions during the release studies, the solubility of CUR in the release medium (0.1% w/v Tween 80 in PBS) was determined and compared with the literature.^25^ An accurately weighed CUR (∼10 mg) was placed in dialysis membrane bags previously activated in PBS and suspended in 10 mL of 0.1% (w/v) Tween 80 in PBS. The system was sealed using plastic clips and placed in an ISF 7100 orbital incubator (Jeio Tech, Ma, USA) at 37 °C with an agitation speed of 100 rpm. One mL of samples was withdrawn at chosen time points (0 h, 4 h, 1 day, 2 days, 3 days, 7 days, 14 days, 21 days, and 28 days) and replaced with fresh media, then quantified by absorbance at a 425 nm wavelength, with a previous calibration curve. The release data were fitted to zero-order, first-order, Hixson–Crowell, Baker–Lonsdale, and Higuchi models, and the regression was determined to evaluate release mechanisms and kinetics. This experiment was carried out in triplicate, and the results were expressed as the mean ± SD.

### 2.7 Mucin Binding Efficiency Study

The mucoadhesive properties of NCs were evaluated indirectly by measuring the absence of mucin in the supernatant of the solution incubated with NCs after its precipitation. Briefly, the NC dispersion (1 mg/mL) was mixed (1:1 v/v) with a previously centrifugated mucin solution (2 mg/mL). Afterward, the mix was incubated at 10 mM PBS pH 7.4 for 60 min and subsequently centrifuged at 13,552*g* for 30 min. The remaining free mucin in the supernatant was determined at 261 nm by UV/visible spectroscopy (LOD = 123.2 μg/mL; LOQ = 373.5 μg/mL) and compared to that of the control sample (without NCs). The pellet was recovered, redispersed in fresh water, and precipitated once more by centrifugation. The final pellet was characterized by DLS and zeta potential to determine its stability and mucin corona formation. Samples were prepared and measured in triplicate, and the data were presented as the mean ± SD.

### 2.8 Mucosal Deposition and Drug Extraction Method

Intestinal segments were extracted from the calf slaughtered for a commercial meat production process. These segments were used due to their high amount of mucin and ease of characterization in this tissue.^26^ The fresh intestinal segments were carefully opened longitudinally, frozen in liquid nitrogen, stored at −20 °C, and defrosted overnight in the refrigerator before use. For the ex vivo experiment, a piece of 3 cm × 3 cm calf small intestine was placed in a Petri dish with wetted soft paper on the bottom. A plastic cylinder of 2 cm in diameter was driven into the mucosal tissue to act as a sample container. In this area, 150 μL of solution of NCs (1 mg/mL) was deposited and incubated in a temperature gradient from 32 to 37 °C for the first 30 min, and a constant temperature of 37 °C until 2 h elapsed. After the incubation time, the tissue was cleaned with abundant water to eliminate the NCs that did not penetrate the mucosal tissue, and it was immersed in 2 mL of acetonitrile for 3 days to extract the penetrated CUR. Subsequently, it was sonicated and centrifuged at 13,552*g*, and the amount in the supernatant was quantified by absorbance at 425 nm using a quartz cuvette. All measurements were conducted in triplicate, and the data were presented as the mean ± SD.

### 2.9 Cell Culture

THP-1 monocyte cell line was purchased from ATCC. To differentiate THP-1 into macrophages, 100 ng/mL of phorbol 12-myristate 13-acetate (PMA) was used on a monocyte cell suspension. Calu-3 human adenocarcinoma epithelial cell line was purchased from ATCC (ATCC HTB-55, LGC Standard, Spain). Human microglia clone 3 (HMC3) was purchased from ATCC.

### 2.10 CUR NC Exposure and Effect on Metabolic Activity and Cell Viability

NC formulations were redispersed in PBS 1× (pH 7.4) before cell exposure. 5 ×10^4^ cells per well were seeded into 48-well plates with PMA-conditioned media for 24 h. After differentiation, the medium was refreshed with RPMI medium. The cells were treated with LPS (100 ng/mL) and/or different concentrations of NCs in RPMI medium (from 500 to 6.25 μg/mL). Cells were exposed to NCs for 24 h to determine the effect on the acute phase of inflammation. Afterward, the supernatants were recovered and stored at −20 °C for further analysis. Culture’s metabolic activity was evaluated using alamarBlue (Thermo Fisher). In brief, a 10% alamarBlue solution was added to the culture and incubated for 4 h at 37 °C (5% CO_2_, humidity). The reduced alamarBlue was determined by analyzing the supernatant fluorescence using a plate reader (λ_ex_ 545 nm, λ_em_ 590 nm).

dsDNA content was measured to evaluate cell viability using PicoGreen assay. Briefly, a known volume of Mili-Q water was added to the cell culture before freezing. The samples were then subjected to cycles of freeze–thaw. PicoGreen assay was performed following the manufacturer’s protocol (PG, Thermo Fisher). The ratio of metabolic activity to dsDNA content was calculated. Both analyses were performed at least in triplicate (*N* ≥ 3).

### 2.11 Cytokine Release Determination by Enzyme-Linked Immunosorbent Assay

THP-1 differentiated macrophages response to NCs at the highest concentration that showed no toxicity in any of the systems tested (50 μg/mL) was measured by enzyme-linked immunosorbent assay (ELISA) according to the manufacturer’s specification (DuoSet ELISA Kits; R&D). Briefly, the three pivotal pro-inflammatory cytokines [tumor necrosis factor-α (TNF-α), IL-1β, and IL-6] were quantified in the recovered culture media after the treatment. Cytokine release was assessed in triplicate (*N* ≥ 3).

### 2.12 NCs Permeability across the Airway Epithelia

The Calu-3 cell model was implemented to mimic the nose-to-brain barrier.^27^ Briefly, 5 × 10^5^ Calu-3 cells per well were seeded on Transwell polyester inserts with a 3 μm pore diameter (Corning Costar). The culture medium was changed every 2–3 days in both compartments until the monolayer was created (1 week, according to the Lucifer yellow assay; a permeability of <2% was used as a threshold to define a tight epithelium). After 1 week in culture, the medium was removed from the apical compartment to create an air–liquid interface (AIL). At day 10 in vitro, the media was changed for the Hanks Balance solution (HBSS, Fisher), and 50 μg/mL of the different NC formulations was added to the upper chamber. At selected time points (1, 2, 4, and 6 h), 100 μL of the supernatant in the bottom chamber was collected and analyzed by fluorescence (λ_ex_: 425/20 nm, λ_em_: 525/20 nm) to determine the amount of CUR crossing the monolayer. The permeability % was calculated with the following equation
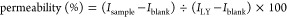
2where *I*_LY_, *I*_sample_, and *I*_blank_ are the fluorescence intensities of the dye, the sample, and the blank (cell-free sample), respectively.

### 2.13 NCs Uptake by HMC3

HMC3 cells were cultured using minimum essential medium supplemented with 10% FBS. 3 ×10^5^ cells/well were seeded in 24 well plates in complete media. After 24 h in culture, a known concentration of 50 μg/mL of raw CUR (CUR dissolved in DMSO) or the NC formulations diluted in media was added to HMC3 cells. Four and 24 h after the treatment, the supernatant was removed, and the cells were washed three times with PBS prior fixation with 4% paraformaldehyde (PFA). Afterward, the PFA was removed, and the cells were washed three times with PBS, and the nuclei were stained with DAPI. The samples were then analyzed in a fluorescence microscope inverted Nikon Eclipse Ti2 (Nikon, Japan). The experiment was performed in triplicate (*n* = 3).

### 2.14 Statistical Analysis

Experimental data were given as the mean ± SD (*N* ≥ 3). The one-way analysis of variance (ANOVA) method followed by the Tukey multiple comparisons test was adopted to evaluate the significance level of the experimental data between groups through Graphpad Prism 9. A *p*-value of 0.05 was selected as the significance level, and all data were marked as * for *p* < 0.05, ** for *p* < 0.01, and *** for *p* < 0.001.

## 3 Results and Discussion

### 3.1 Preparation and Characterization of CUR NCs with Polymeric Surface Layer

**Figure 1:**
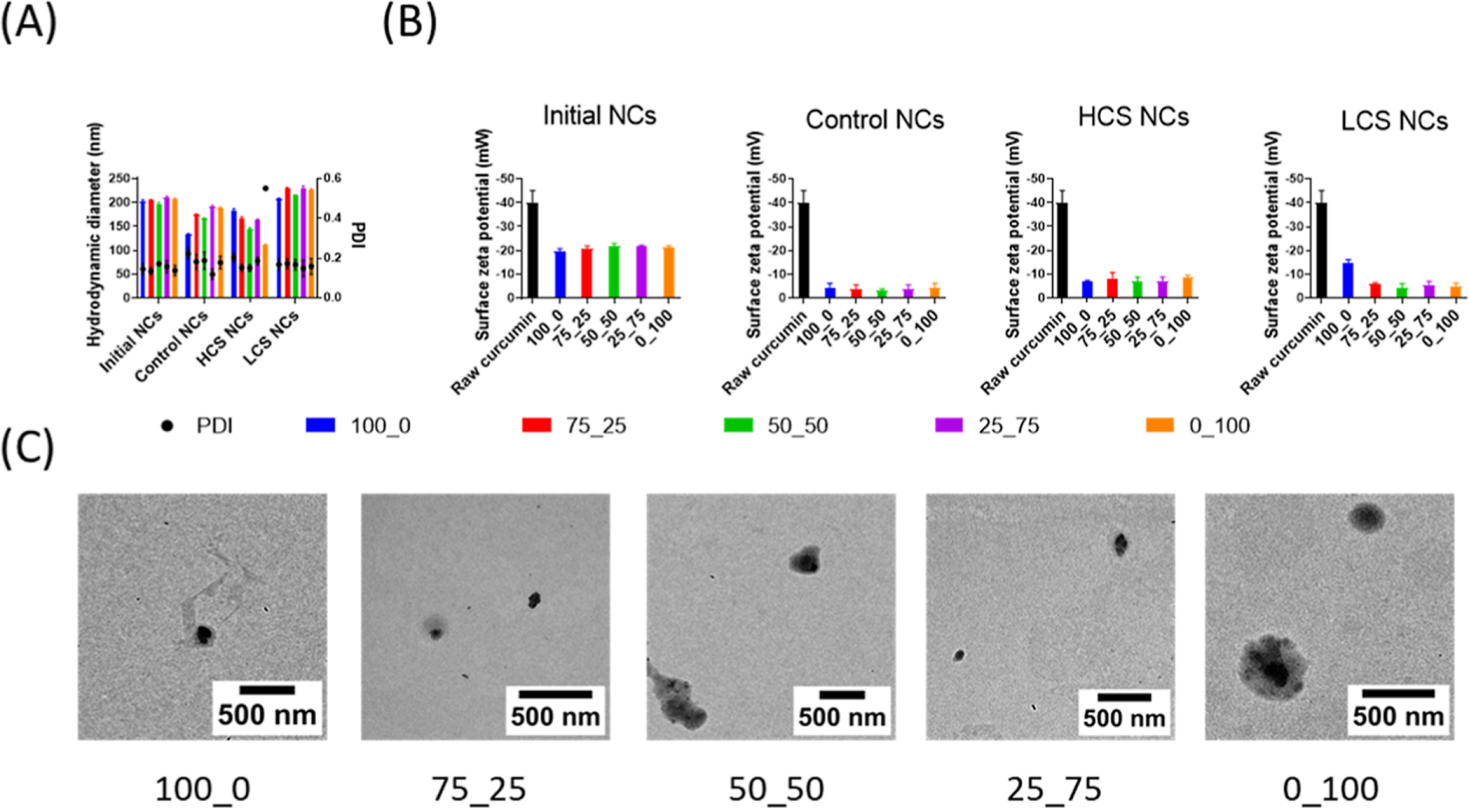
CUR NCs preparation and characterization. Initial NCs correspond to NC formulations before polymerization. (A) Hydrodynamic diameter (nm) (bars) and PDI (circles) measured by intensity distribution in DLS. (B) Surface zeta potential. (C) TEM micrographs of initial CUR NCs. Data represented as the mean ± SD; *n* = 3.

A media milling device operates by dispersing the material (in this case, NCs) in a liquid medium along with grinding media (e.g., ceramic beads). The milling process involves the agitation of the mixture, causing the grinding media to collide with the material, thereby reducing its size through mechanical attrition.^9^ The initial CUR NCs were stabilized using various poloxamer: poloxamer diacrylate ratios, thus modulating the availability of acrylic reactive groups on the NCs’ surface. A consistent average hydrodynamic size of ∼200 ± 5 nm was observed across all formulations (Figure 1A), indicating an optimal mucopenetrating size for all the NCs.^22^ It was found that these NCs remained stable under the purification conditions used, as they maintained their hydrodynamic size (∼200 nm) and PDI (∼0.2) (Figure S1 of the Supporting Information). After polymerization, this set of nanosystems was taken as the control group (Control NCs). Furthermore, two additional polymerization strategies were conducted, incorporating PEG-acrylate or PEG-diacrylate into the previous formulations, pursuing modulation of the chemical affinity of the polymer layer with mucin, and boosting their cage protective effect. These formulations were called LCS NCs and HCS NCs, respectively.

Compared with initial NCs, both Control NCs and HCS NCs displayed a size reduction after polymerization. This size change could be attributed to a more compact arrangement of the surrounding polymer layers, resulting in a decrease in hydrodynamic size.^28,29^ In contrast, LCS NCs increased in size after polymerization, except for the 100_0 formulation, which was stabilized only with the nonreactive poloxamer. This result suggests that introducing PEG-acrylate effectively led to a loosely cross-linked polymer layer on the NCs’ surface, consequently increasing the hydrodynamic size. Notably, z-potential measurements revealed that all NC variations presented a more neutral nature (∼−5 mV) than raw CUR (∼−40 mV) (Figure 1B). Several studies indicate that slightly negatively charged nanoparticles are more likely to reduce interaction with the mucosal barrier.^22^ This is because the main component of the barrier, mucin, has a slightly negative charge. Thus, a highly negative nanoparticle would be repelled and a highly positive one would adhere. Therefore, it is hypothesized that the synthesized NCs have an optimal charge to maintain a low interaction with mucin. Additionally, slightly negative NCs have the longest half-lives in circulation; therefore, they are often preferred for prolonged systemic circulation and enhanced drug delivery to target sites.^30^

Is worth noting that despite this low z-potential, all the NCs show long-term stability (Figures S2–S4), confirming the polymer layer’s steric protection and successful surface modification.^31^ The NCs’ stability was assessed through DLS at 37, 4 °C, and RT, simulating physiological, storage, and application temperatures, respectively (Figures S2–S4). Thus, this demonstrates that all NCs remain stable for at least 30 days, as evidenced by minimal changes in size and PDI. TEM imaging of initial NCs (Figure 1C) corroborated their starting structure, showcasing a distinct CUR core (∼200 nm) enveloped by a polymeric coating.

Furthermore, FTIR, ^1^H NMR, and DSC (Figures 2 and S5–S12) analyses confirmed the successful incorporation of the polymeric components into the CUR NCs.

**Figure 2:**
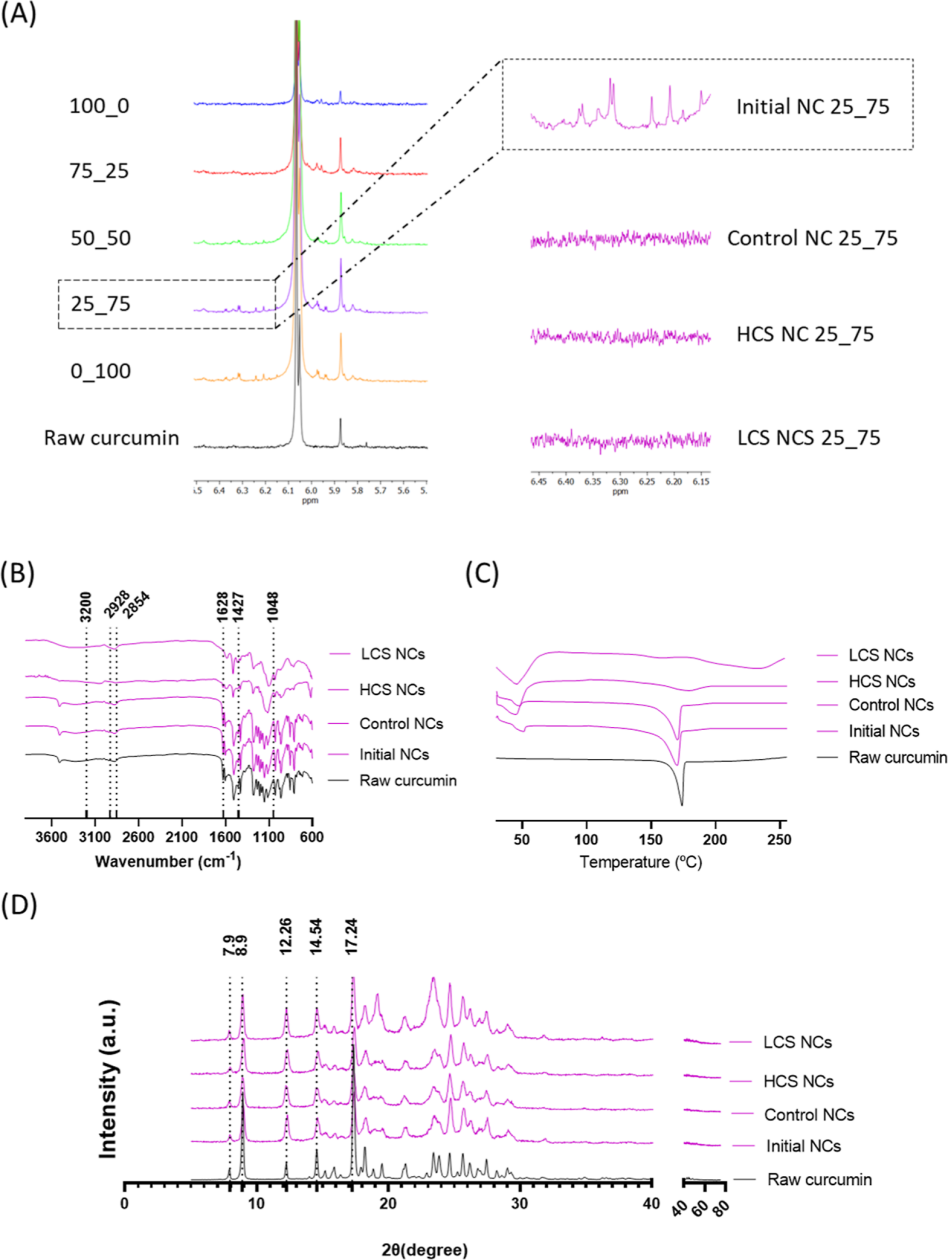
CUR NCs physicochemical characterization. (A) ^1^H NMR of initial NCs with different nonacrylated: acrylated stabilizer weight ratios is solved in DMSO-*d*_9_. Zoom represents initial NCs 25_75, Control 25_75NCs, HCS 25_75NCs, and LCS 25_75NCs, sam (B) FTIR spectra of the 25_75 NC variations, (C) DSC curves of the different 25_75 NCs variations, heated in a nitrogen atmosphere at a stepping rate of 10 °C/min over a temperature range of 0–200 °C with an empty pan as reference, (D) XRD of the 25_75 NC variations. Complete analysis of ^1^H NMR can be found in the Supporting Information, Figures S5–S10.

Through comprehensive analysis using ^1^H NMR (Figures 2A and S5–S10) and FTIR techniques (Figures 2B and S11), it was observed that the characteristic signals corresponding to CUR remained unchanged after the milling process, indicating the preservation of its chemical composition. Additionally, new peaks originating from the polymers were identified in the spectra, at 6.3 ppm band from the double bonds of the PEG-diacrylates and a 3.35 ppm band from the terminal methyl group of the PEG. Further confirmation of the presence of the polymers was obtained through DSC (Figure S12), since the polymer bands are observed at ∼50 °C, as seen in the DSC of the control materials (Figure S13).

As observed in the inlets in Figure 2A, ^1^H NMR analysis provided further evidence of the polymer layer formation, showing the disappearance of vinyl proton signals (δ 5.5–6.5 ppm) after polymerization. The absence of these signals indicates that the polymerization process effectively took place, transforming the structure and composition of the NCs. The mechanism underlying this transformation involves the initiation of polymerization, catalyzed by initiators present in the reaction mixture (poloxamer and poloxamer diacrylate). As polymerization progresses, monomeric units undergo chain propagation, leading to the growth of polymer chains and the encapsulation of NCs within a polymer network.

Figure 2B shows the FTIR spectra for raw CUR, 25_75 initial NCs, 25_75 control NCs, 25_75 HCS NCs, and 25_75 LCS NCs. FTIR spectra illustrate characteristic signals at 1628 cm^–1^ primarily attributed to the overlapping stretching vibrations characteristic of alkene (C=C) and carbonyl (C=O) functionalities. The asymmetrical and symmetrical stretch modes of the (C=C) groups occur at 2928 and 2854 cm^–1^, respectively. The infrared spectrum of CUR reveals stretching vibrations at 3200–3500 cm^–1^, corresponding to the O–H groups, aromatic C=C stretching vibrations at 1427 cm^–1^, and a high-intensity band at 1512 cm^–1^. These peaks are associated with mixed vibrations encompassing stretching carbonyl bond stretching (ν (C=O)), in-plane bending vibrations of aliphatic (δ CC–C, δ CC=O), and in-plane bending vibrations of aromatic (δ CC–H) configurations of keto and enol forms. Moreover, the C–O stretch of C–O–H groups is appreciated at 1048 cm^–1^. Additionally, stretching vibrations of aromatic (ν CC) bonds of both keto and enolic forms of CUR are evident. Therefore, CUR’s structure can be confirmed in all NCs.

Using DSC, we could discern the characteristic melting peaks of the polymers. After control experiments were conducted with only the polymers, similar trends were observed in the curves. Furthermore, the overlapping of the melting peaks is evident when there are mixtures of different polymers. Therefore, we confirm that NCs contain polymers. Through DSC, bands of both CUR and the polymer can be observed. For the raw CUR sample, only one band is shown at 175 °C, corresponding to the melting point of the drug. As for the samples of 25_75 initial NCs, 25_75 control NCs, 25_75 HCS NCs, and 25_75 LCS NCs, bands between 40 and 50 °C are observed, corresponding to the polymeric stabilizers as confirmed by control measurements of these polymers (Figure S13). Interestingly, very faint CUR bands are observed at 175 °C for these NCs. This reduction in intensity can be attributed to polymer melting, which leads to thermally induced amorphization as temperature increases during the DSC experiment, accompanied by CUR’s dissolution, resulting in an overall intensity decrease.^32^

XRD analysis was performed to verify that CUR’s crystallinity was maintained in all NCs. XRD analysis of samples was done over a broad angle range (2θ = 5–80°). The powder X-ray diffractograms of raw CUR, 25_75 initial NCs, 25_75 control NCs, 25_75 HCS NCs, and 25_75 LCS NCs dried powders are shown in Figure 2C. The five characteristic peaks of CUR appeared at diffraction angles 2θ at 7.96, 8.90, 12.26, 14.54, and 17.24°.^33^ The diffraction patterns of the NCs obtained after milling and polymerization showed an identical pattern to that of raw CUR, indicating its presence as a crystalline form while ruling out the potential formation of polymorphic forms of the drug.

These findings collectively illustrate the alterations in size, surface properties, and structure postpolymerization. Hence, it underscored the successful modification and incorporation of polymers onto the NCs’ surface, potentially enhancing their stability, preventing the Ostwald ripening phenomenon, and highlighting their characteristics for optimized drug delivery applications.

### 3.2 Drug Content and In Vitro Release Profiles

**Figure 3:**
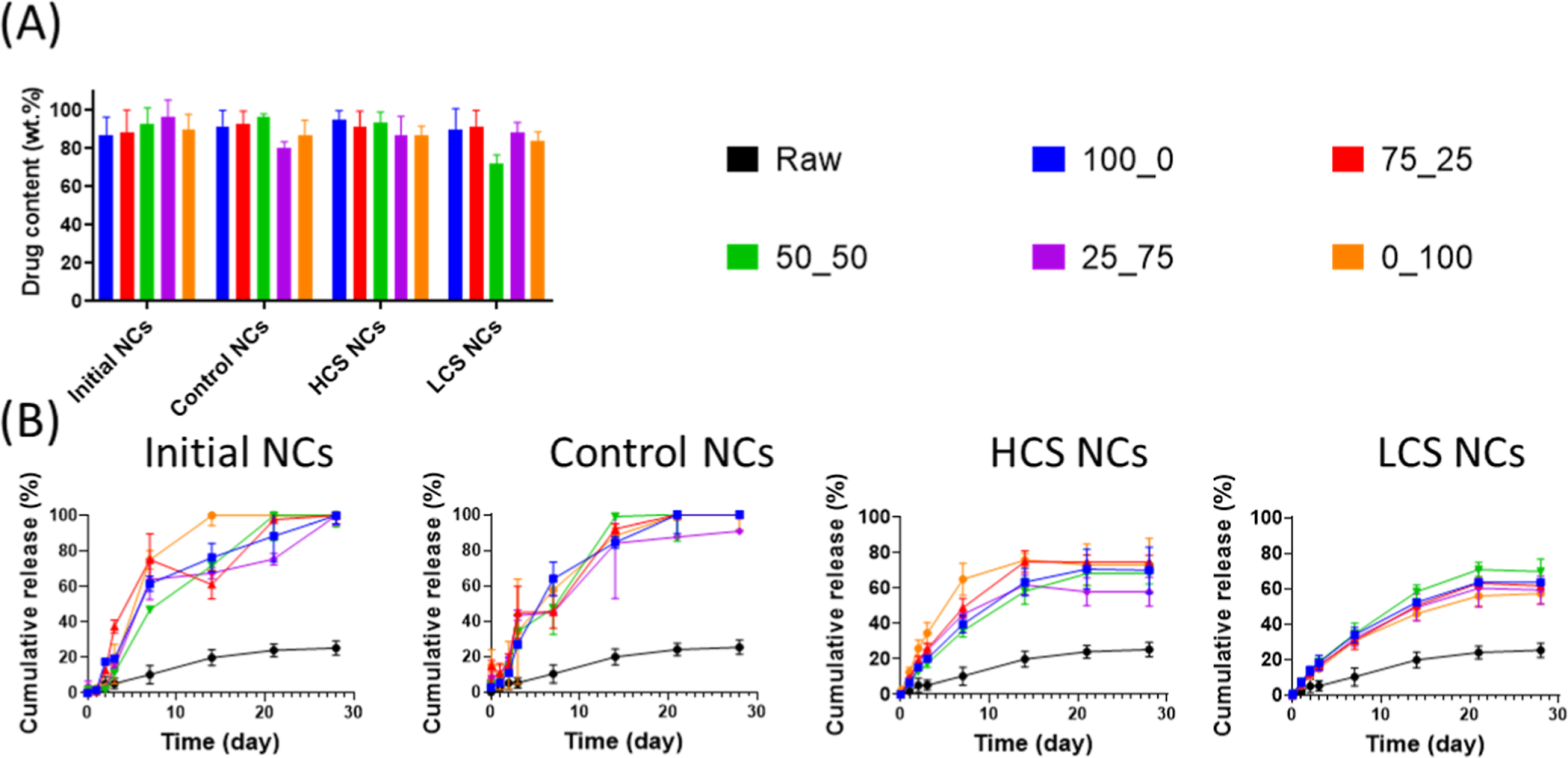
In vitro controlled release of CUR from NCs. (A) Drug content and (B) release kinetics at 37 °C in phosphate buffer pH 7.4 10 mM containing 1% w/v of Tween 80. Data represented as the mean ± SD; *n* = 3.

The drug content within all NC formulations was notably high (e.g., >80% of the total composition), displaying consistency across the different formulations without any distinct observable trend (Figure 3A). Comparatively, this drug content surpasses enormously the levels typically obtained with polymeric nanoparticles.^34^ This holds significant promise for potential applications, suggesting the feasibility of reducing doses and subsequently mitigating cytotoxicity and side effects.^35^

Regarding drug release profiles, experiments conducted in PBS with 1% Tween 80 revealed that the NCs could deliver approximately 80% of their cargo over 30 days (Figure 3B). The same trends were observed in the NCs incubated in 10 mM PBS pH 7.4 with 1% ascorbic acid to prevent drug degradation.^24^ Furthermore, under these conditions, an approximately 20% higher release was observed for HC NCs and LC NCs, suggesting that CUR degradation was potentially prevented in these NCs within this medium (Figure S14). It should be noted that the initial NCs 75:25 formulation showed a decrease in the average cumulative release on day 14. We identified one outlier at days 7 and 14 in one of the samples (*n* = 1), likely due to CUR adhesion to the dialysis membrane or a minor experimental error. However, since no statistically significant differences were found between these two samples, as they fall within the standard variation, we decided to include all samples (*n* = 3) in the analysis.

The release mechanisms and kinetics of the NCs were evaluated. The release data were fitted to zero order, first order, Hixson and Crowell, Baker and Lonsdale, and Higuchi models to evaluate release mechanisms and kinetic parameters (Tables S1–S5). The release data were well fitted with Baker and Lonsdale models with *R*^2^ values higher than 0.900, which indicates that drug is released from spherical monolithic drug delivery devices, in which the active pharmaceutical ingredient is initially dispersed throughout an inert diffusion matrix.^36^

Notably, distinct kinetics were observed among the different NC formulations, as the constant k values of the fitted Baker and Lonsdale models are different (Table S6). Both initial NCs and Control NCs exhibited the highest *k* values, indicating faster release kinetics. In contrast, HCS and LCS NC variations showed slower release kinetics, indicating a more sustained and controlled cargo release. It is hypothesized that the release rate of CUR was slower in these formulations due to the thicker polymerized surface, leading to a slower drug diffusion process.

This differential release behavior among the NC variations represents an opportunity for tailored drug delivery systems.^37^ Variations in release rates can be strategically utilized based on specific therapeutic requirements. Faster release kinetics might benefit immediate therapeutic action, while slower and sustained release could help long-term or targeted treatments. Noyes and Whitney’s equation posits that the dissolution rate of a solid material is proportional to its surface area and the concentration gradient.^38^ In NCs, the markedly increased surface area-to-volume ratio promotes faster dissolution by facilitating more efficient interactions with the solvent, following this principle.

### 3.3 Mucin Interactions and Mucosal Tissue Penetration

**Figure 4:**
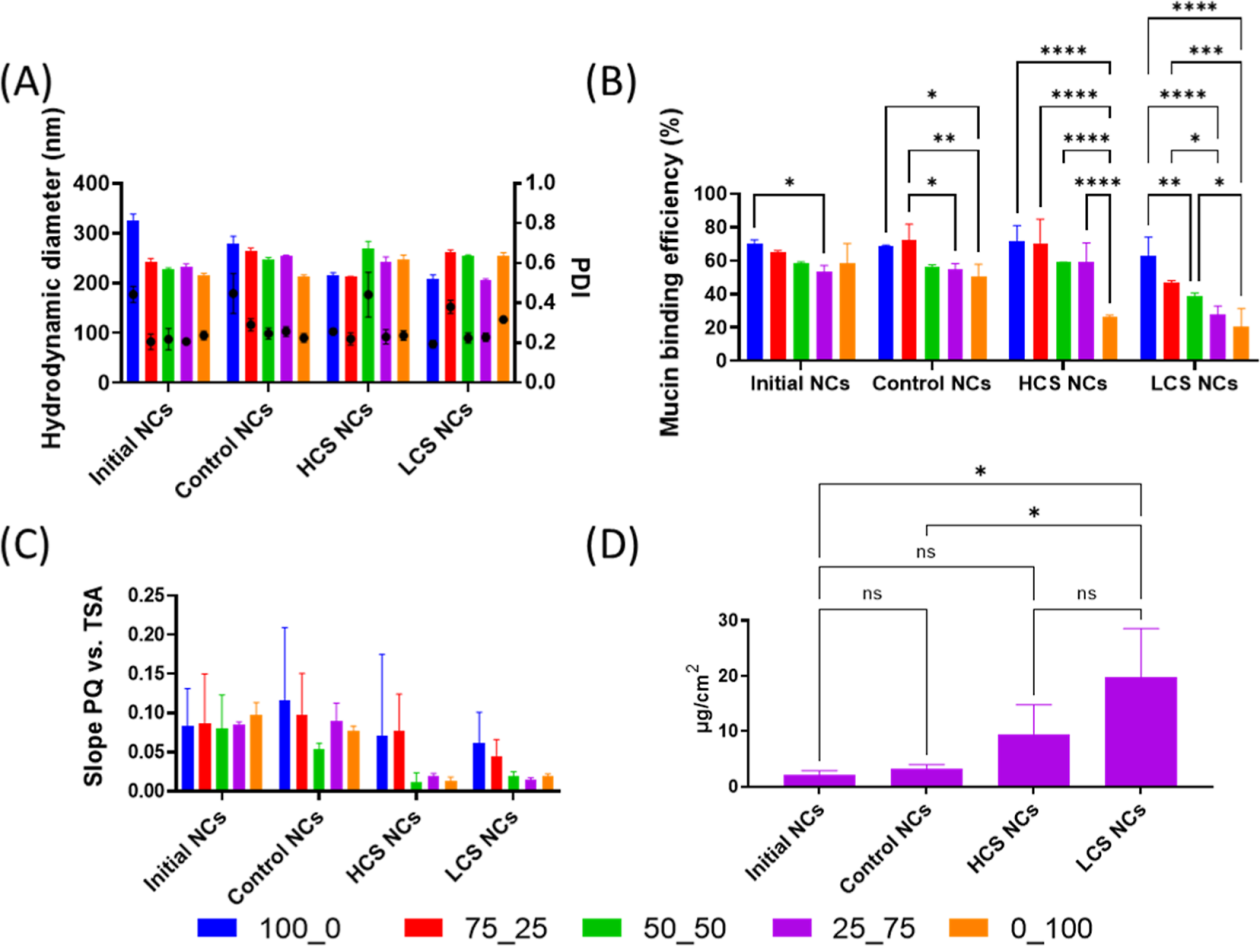
Mucin and mucosal interaction studies. (A) NC dispersions (1 mg/mL) after incubating with a previously centrifugated mucin solution (2 mg/mL), (B) mucin binding efficiency of the NCs measured at 10 mM PBS pH 7.4 for 60 min, (C) Rose Bengal test for the NC formulations, and (D) 25_75 NC penetration in mucosal tissue. Data represented as the mean ± SD; *n* = 3. Tukey multiple comparisons test was used *p < 0.05; **p < 0.01. ***p < 0.005; ****p < 0.001 compared with the control (CTRL) group.

Incubation of the NCs with a mucin solution revealed a slight increase in the size of approximately 20–50 nm without any distinct trend observed among the different NC variations (Figure 4A). Similarly, alterations in the zeta potential were noted in the NCs. These changes in size and zeta potential following mucin exposure imply the formation of a protein corona, indicating interactions between mucin and the NCs.^39^

The characterization of mucin binding efficiency, as observed in Figure 4B, portrayed notable differences between the initial NCs and those postpolymerized. After polymerization, significant changes were observed, particularly indicating a trend in which an increase in the content of the poloxamer diacrylate led to a reduction in the mucin binding. Notably, the LCS NCs exhibited more substantial changes than the HCS and Control NCs, implying a potentially more efficient reduction in interaction with mucin for the loosely cross-linked topology. Numerous researchers have documented that surface hydrophilicity plays a pivotal role in these interactions, influencing protein corona formation and subsequent mucosal tissue penetration.^18^ The Rose Bengal test (Figure 4C), although limited due to the high deviations among different NCs, supports this evidence. A reduced hydrophobicity was observed in the HCS and LCS NC formulations with a higher content of the reactive poloxamer, which perfectly aligns with the findings from mucin experiments. This result suggests that a more hydrophilic surface might reduce interactions with the hydrophobic mucin protein. Furthermore, mucosal tissue penetration studies characterized with calf small intestine demonstrated that HCS and LCS NCs are more effective in passing through the mucus barrier than the other systems assessed, although no significant differences were observed between both formulations (Figure 4D).

Altogether, the high contents of poloxamer diacrylate in polymerized systems seem to influence mucin interactions, potentially reducing mucin binding efficiency significantly. However, at this point, it is worth mentioning that NCs with the highest ratio of reactive poloxamer (0_100) have the highest size polydispersities. Therefore, 25_75 NC formulations appear more promising for effectively stabilizing and reducing mucin interaction. Besides, the LCS NC variation particularly stands out, showing potential as a candidate for reduced interaction with mucin and improved tissue penetration, which could hold significance in drug delivery applications. Consequently, further biological analyses were performed over the LCS 25_75 NC formulation.

### 3.4 In Vitro Cell Studies

Addressing the global impact of CNS disorders is challenging from a pharmacological point of view because of the so-called blood–brain barrier, which shields the brain and spinal cord.^40^ In this particular context, the nose-to-brain path is a noninvasive approach, which allows the direct transport of drugs through olfactory and trigeminal nerve pathways. However, drug absorption and permeability through the nasal epithelium are still challenges to overcome for successful drug delivery to the brain.^16^ Thus, the design and development of new formulations capable of efficiently surpassing the nasal barriers, ensuring adequate drug bioavailability in the brain parenchyma, are still desired.

**Figure 5:**
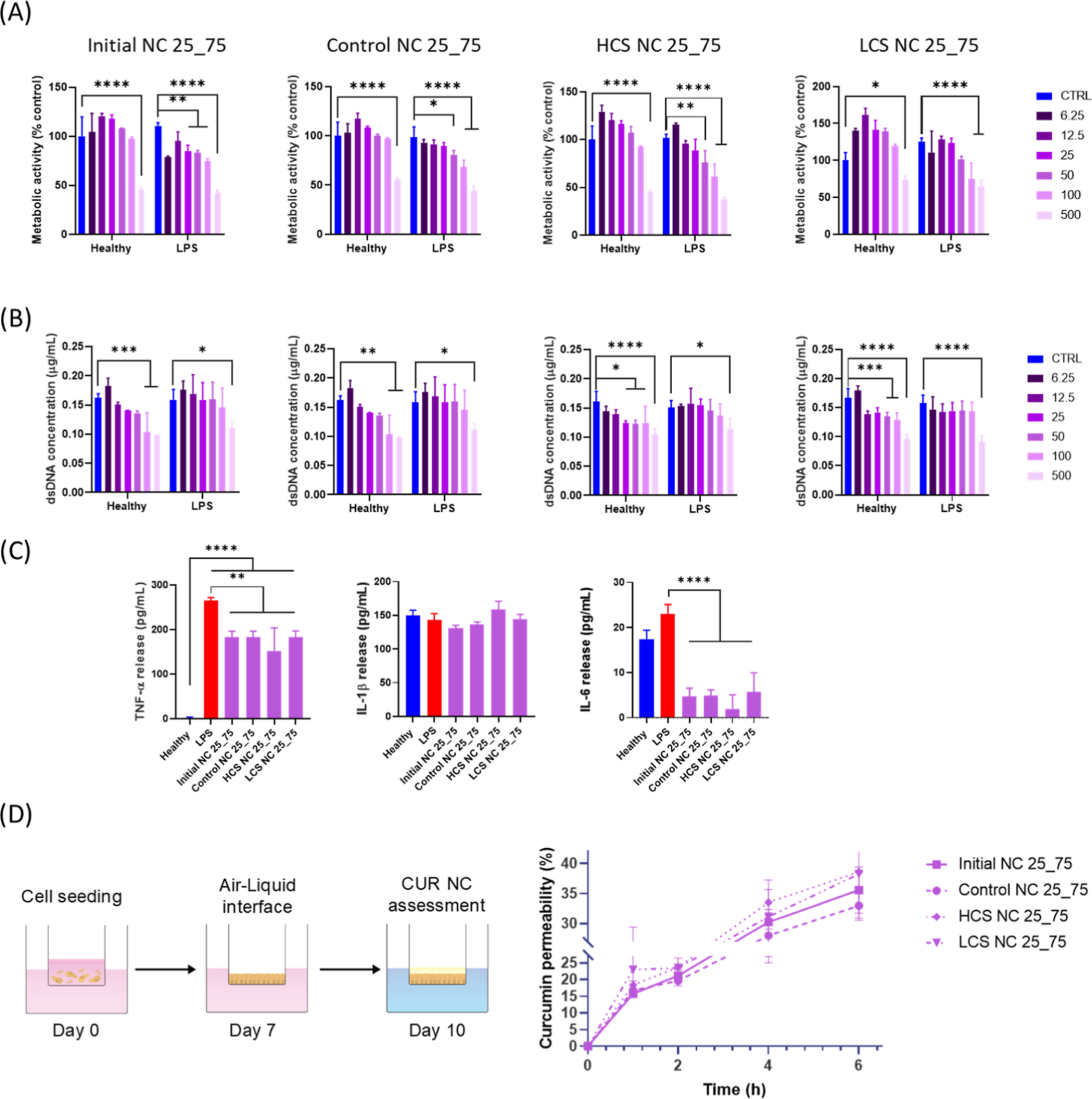
In vitro performance of CUR NCs. (A) Effect of the different CUR NC formulations at doses from 6.25 to 500 μg/mL on the metabolic activity of THP-1-derived macrophages with or without a 100 ng/mL lipopolysaccharide (LPS) pretreatment determined by alamarBlue and normalized to dsDNA content determined by PicoGreen; (B) dsDNA content determined by PicoGreen after 24 h of exposure, (C) effect on the cytokine release from THP-1 pre-exposed to 100 ng/mL LPS, measured as the concentration in the supernatant after the treatment. Data obtained from *n* > 3. Mean ± SD, double (A,B), or single (C) ANOVA. Tukey multiple comparisons test was used *p < 0.05; **p < 0.01. ***p < 0.005; ****p < 0.001 compared with the control (CTRL) group, (D) CUR permeability over time through a Calu-3 monolayer. Schematic view of the assay performed and NCs ability to cross the monolayer membrane using a 50 μg/mL concentration over time. CUR permeability expressed as % of the total CUR capable of crossing the monolayer determined by fluorescence (λ_ex_: 425/20 nm, λ_em_: 525/20 nm). Data obtained from *n* > 3. Mean ± SD.

Moreover, inflammation plays a pivotal role in the onset of most CNS disorders.^41^ Macrophages and microglia are the key players in initiating, maintaining, and resolving the inflammatory response in the CNS. Thus, it is crucial to study the potential effect of our NCs on macrophages to ensure a suitable immune response. THP-1 is a valuable and reliable tool for studying immune and inflammatory responses when exposed to various treatments.^41^ THP-1 is an immortalized monocyte-like cell line derived from the blood of an acute monocytic leukemia patient.^41^ Hence, the metabolic activity (normalized to dsDNA content) in the presence of NCs was investigated using THP-1-derived macrophages. Moreover, the effect of NCs on THP-1 was assessed with or without exposure to a 100 ng/mL LPS concentration to study their impact on inflammation-activated cells. A clear trend is observed in Figure 5A, indicating a dose-dependent reduction in metabolic activity. This may relate to CUR’s bioactive properties, as it is a potent inhibitor of various cellular processes, including metabolic activity.^42^ Interestingly, only the highest dose of 500 μg/mL caused a significant reduction in the metabolic activity in all of the NCs tested on healthy THP-1. On the contrary, LPS-pretreated THP-1 cells seem to be more sensitive to NC treatment. However, only the treatment with 500 μg/mL NC doses reduced metabolic activity below 70%. A decrease in the dsDNA content was evident in cells treated with the two higher concentrations of NCs (Figure 5B), indicating an acute cytotoxic effect of the higher dose. Conversely, doses of 100 μg/mL showed a slight decrease in dsDNA content or no differences compared to healthy cells, indicating an appealing performance in terms of cytotoxicity.

The impact of CUR NCs on the release of pro-inflammatory cytokines was also investigated and is shown in Figure 5C. The focus was set on the three pivotal cytokines mediating acute inflammatory responses: TNF-α, IL-1β (interleukin-1β), and IL-6 (interleukin-6). The experiment involved comparing the cytokine release from cells treated with NCs to that from healthy cells and cells stimulated with LPS, a known inducer of inflammatory responses. First, the effect of NCs on healthy THP-1 was assessed (Figure S15). All the NCs tested induced a higher release of TNF-α than healthy cells. However, the release was considerably lower than the one induced by LPS treatment. A similar pattern was observed for IL-1β and IL-6, proving the safety of NCs in vitro at the dose tested (50 μg/mL). Interestingly, all of the assessed formulations ameliorated the LPS effect, exhibiting a significantly lower TNF-α release than the cells from the LPS group (Figure 5C).

As observed, all NCs exhibited release levels comparable to those of healthy and LPS-treated cells. IL-1β is a key inflammatory mediator, and this finding suggests that NCs have a minimal impact on their release. This specificity of action is valuable for developing targeted therapies, ensuring safety by avoiding excessive immune suppression. The lack of significant modulation of IL-1β release provides insight into the nuanced immunomodulatory effects of NCs.

Notably, in the context of IL-6 release, cells treated with NCs demonstrated lower release levels than healthy and LPS-treated cells, suggesting a potential inhibitory effect on this cytokine. IL-6 is a pro-inflammatory cytokine associated with immune responses, and its reduced release indicates a possible anti-inflammatory effect exerted by the NCs.^42^ Interestingly, it has been reported that CUR significantly reduces circulating IL-6 concentrations.^43^ However, a better design of the clinical trials and larger populations are still needed to determine the real therapeutic impact of its anti-inflammatory effect.

These findings indicate that despite surface modifications, there was no significant alteration in the activity of CUR, exhibiting a promising therapeutic potential to ameliorate inflammatory-related disorders.

The study of drug diffusion when administered nasally remains a challenge both in vivo and at a clinical level.^44^ Hence, the use of relevant in vitro models to assess drug permeability through the nasal epithelium is an appealing approach to elucidating the transport of new therapeutics. Herein, a transwell in vitro model of the Calu-3 cell line was used to study drug transport.^45^ First, the membrane integrity of the Calu-3 monolayer cultured under AIL was checked by the Lucifer yellow test, which measures the paracellular transport of the molecule (Figure S16). After 10 days in culture, the Lucifer yellow permeability was lower than 2%, confirming the integrity of the epithelium and its suitability for drug transport studies.^27^ The NCs’ ability to cross the monolayer membrane was then assessed using a 50 μg/mL concentration (Figure 5D). All the tested NCs could efficiently cross the cell membrane, with an approximate 40% penetration over a 6 h period. However, no statistically significant differences were observed among the NC formulations in this aspect. This lack of distinction may be attributed to the intrinsic hydrophilic nature of all NCs, a characteristic induced by the stabilizing agents used. Moreover, CUR-related fluorescence was not observed in the monolayer or the transwell membrane under fluorescence microscopy analysis (data not shown), suggesting the ability of the NCs to cross the barrier efficiently due to their small hydrodynamic size. These results agree with previous reports assessing CUR NCs’ size-dependent ability to cross the Calu-3 monolayer.^46^ Altogether, these results suggest the potential for efficient CUR delivery through the nose-to-brain path of the NCs tested.

Furthermore, the ability of our formulations to be taken up by HMC3 was assessed. HMC3 was chosen to try to mimic the scenario when the NCs would reach the brain parenchyma. The uptake of the NCs and the raw CUR can be appreciated after 4 h (Figure S16) and 24 h (Figure S17) when compared with the control cells that did not receive any treatment. It is worth noting the lower number of viable cells present in the group treated with free CUR (CUR dissolved in DMSO) compared to those exposed to the different formulations of NCs, supporting the potential safety of the nanoformulation. Moreover, the relative uptake was higher after 24 h (Figure S17) than after 4 h of exposure (Figure S16). At 24 h, a similar trend in the number of viable cells was observed in all the NC formulations, being notably higher than in the raw CUR-treated group. Altogether, all of the NCs tested exhibited a suitable performance over three different cell lines, making them promising candidates for future experiments on more complex models and for preclinical testing.

## 4 Conclusions

In this study, we explored the surface polymerization of PEG on drug NCs as an innovative strategy for surface chemistry modulation to enhance targeted delivery. The investigation revealed substantial alterations in the properties and behaviors of these modified NCs, offering insights into their potential for optimized drug delivery systems.

By employing various analytical techniques such as DLS, TEM, FTIR, NMR, DSC, and XRD, this study confirms successful polymer incorporation onto NC surfaces while preserving CUR’s chemical composition. These modifications result in size, surface, and structural changes postpolymerization, enhancing the stability and solubility of NCs. Moreover, in vitro drug release studies revealed differential release behavior among NC variations, offering opportunities for tailored drug delivery systems to meet specific therapeutic needs. In vitro studies provided intriguing observations regarding the mucin interaction and mucosal tissue penetration. After polymerization, variations in PEGylation significantly influenced mucin binding efficiency, with LCS NCs exhibiting the most substantial reduction in interaction with mucin. Therefore, surface hydrophilicity emerged as a crucial factor, potentially reducing the affinity with mucin. Furthermore, the tissue penetration analysis revealed that both HCS and LCS NCs exhibited an enhanced ability to pass through the mucosal barrier, presenting promising characteristics for improved drug delivery to the brain.

Cell studies of CUR NCs in THP-1-derived macrophages unveiled a concentration-dependent decrease in cellular metabolic activity. Notably, only the highest concentration decreased the metabolic activity and dsDNA below 70%, exhibiting a promising profile in terms of the in vitro compatibility of the tested NCs.

The investigation into the influence on pro-inflammatory responses demonstrated that while the NCs unmistakably exerted an inhibitory effect on the LPS-induced inflammatory response, they also showcased nuanced immunomodulatory effects. Surprisingly, no significant differences in bioactivity were observed, even when the surface was altered. These observations indicate that despite surface modifications allowing for targeted interactions or reduced interactions with specific biological barriers, the bioactive compounds of the NCs remain unaffected. Furthermore, all of the systems showed an interesting ability to cross biological barriers in a relevant in vitro model of the airway epithelia. Nonetheless, the potential exhibited by the NCs needs further investigation to confirm these properties in a relevant in vivo model of nose-to-brain delivery.

This comprehensive analysis underscores the potential of polymerized NCs as highly loaded nanovectors in revolutionizing targeted drug delivery. The findings suggest that the modifications induced by polymerization offer a means to enhance drug loading, reduce mucin interactions, and potentially tailor the release kinetics for more effective and targeted drug delivery. The outcomes of this study lay the foundation for further exploration and optimization of surface chemistry in NCs for enhanced drug delivery applications, offering a promising pathway toward more efficient and targeted treatments for neurological disorders and other conditions that require brain-specific therapies.

## Supporting Information Available

Fitting of cumulative CUR release curves; comparison of rate constants; NC stability studies; hydrodynamic size before and after freeze-drying; ^1^H- and ^13^C NMR spectra of CUR, poloxamer diacrylate, poloxamer, and different NC formulations; FTIR spectra and DSC curves of different NC variations; CUR release kinetics in 10 mM PBS 7.4 and 10 mM PBS 7.4 with 1% ascorbic acid; effect on the (A) TNFα, (B) IL-1β, and (C) IL-6 release from THP-1-derived macrophages; Lucifer yellow assay; CUR-based formulations uptake after 4 h exposure on HMC3; and micrographs of HMC3 cells after 4/24 h incubation with different formulations (PDF)

## References

[ref1] Couillaud B. M.; Espeau P.; Mignet N.; Corvis Y. State of the Art of Pharmaceutical Solid Forms: from Crystal Property Issues to Nanocrystals Formulation. ChemMedChem 2019, 14 (1), 8–23. 10.1002/cmdc.201800612. 30457705

[ref2] Jarvis M.; Krishnan V.; Mitragotri S. Nanocrystals: A perspective on translational research and clinical studies. Bioeng. Transl. Med. 2019, 4 (1), 5–16. 10.1002/btm2.10122. 30680314 PMC6336669

[ref3] Mohammad I. S.; Hu H.; Yin L.; He W. Drug nanocrystals: Fabrication methods and promising therapeutic applications. Int. J. Pharm. 2019, 562, 187–202. 10.1016/j.ijpharm.2019.02.045. 30851386

[ref4] Müller R. H.; Peters K. Nanosuspensions for the formulation of poorly soluble drugs: I. Preparation by a size-reduction technique. Int. J. Pharm. 1998, 160 (2), 229–237. 10.1016/S0378-5173(97)00311-6.

[ref5] Bhalani D. V.; Nutan B.; Kumar A.; Singh Chandel A. K. Bioavailability Enhancement Techniques for Poorly Aqueous Soluble Drugs and Therapeutics. Biomedicines 2022, 10 (9), 2055 10.3390/biomedicines10092055. 36140156 PMC9495787

[ref6] Samineni R.; Chimakurthy J.; Konidala S. Emerging Role of Biopharmaceutical Classification and Biopharmaceutical Drug Disposition System in Dosage form Development: A Systematic Review. Turk. J. Pharm. Sci. 2022, 19 (6), 706–713. 10.4274/tjps.galenos.2021.73554. 36544401 PMC9780568

[ref7] McGuckin M. B.; Wang J.; Ghanma R.; Qin N.; Palma S. D.; Donnelly R. F.; Paredes A. J. Nanocrystals as a master key to deliver hydrophobic drugs via multiple administration routes. J. Controlled Release 2022, 345, 334–353. 10.1016/j.jconrel.2022.03.012. 35283257

[ref8] Möschwitzer J. P. Drug nanocrystals in the commercial pharmaceutical development process. Int. J. Pharm. 2013, 453 (1), 142–156. 10.1016/j.ijpharm.2012.09.034. 23000841

[ref9] Zhang C.; Jahan S. A.; Zhang J.; Bianchi M. B.; Volpe-Zanutto F.; Baviskar S. M.; Rodriguez-Abetxuko A.; Mishra D.; Magee E.; Gilmore B. F.; Singh T. R. R.; Donnelly R. F.; Larrañeta E.; Paredes A. J. Curcumin nanocrystals-in-nanofibres as a promising platform for the management of periodontal disease. Int. J. Pharm. 2023, 648, 123585 10.1016/j.ijpharm.2023.123585. 37952560

[ref10] Fuster M. G.; Wang J.; Fandiño O.; Víllora G.; Paredes A. J. Folic Acid-Decorated Nanocrystals as Highly Loaded Trojan Horses to Target Cancer Cells. Mol. Pharmaceutics 2024, 21 (6), 2781–2794. 10.1021/acs.molpharmaceut.3c01186. PMC11151209 38676649

[ref11] De Roo J.; Van den Broeck F.; De Keukeleere K.; Martins J. C.; Van Driessche I.; Hens Z. Unravelling the Surface Chemistry of Metal Oxide Nanocrystals, the Role of Acids and Bases. J. Am. Chem. Soc. 2014, 136 (27), 9650–9657. 10.1021/ja5032979. 24945901

[ref12] Pinelli F.; Saadati M.; Zare E. N.; Makvandi P.; Masi M.; Sacchetti A.; Rossi F. A perspective on the applications of functionalized nanogels: promises and challenges. Int. Mater. Rev. 2023, 68 (1), 1–25. 10.1080/09506608.2022.2026864.

[ref13] Udabe J.; Tiwari N.; Picco A.; Huck-Iriart C.; Escudero C.; Calderón M. Multi-hierarchical nanoparticles with tunable core by emulsion polymerization processes. Eur. Polym. J. 2023, 201, 112566 10.1016/j.eurpolymj.2023.112566.

[ref14] Ulbrich K.; Holá K.; Šubr V.; Bakandritsos A.; Tuček J.; Zbořil R. Targeted Drug Delivery with Polymers and Magnetic Nanoparticles: Covalent and Noncovalent Approaches, Release Control, and Clinical Studies. Chem. Rev. 2016, 116 (9), 5338–5431. 10.1021/acs.chemrev.5b00589. 27109701

[ref15] Shen G.; Xing R.; Zhang N.; Chen C.; Ma G.; Yan X. Interfacial Cohesion and Assembly of Bioadhesive Molecules for Design of Long-Term Stable Hydrophobic Nanodrugs toward Effective Anticancer Therapy. ACS Nano 2016, 10 (6), 5720–5729. 10.1021/acsnano.5b07276. 27223166

[ref16] Formica M. L.; Real D. A.; Picchio M. L.; Catlin E.; Donnelly R. F.; Paredes A. J. On a highway to the brain: A review on nose-to-brain drug delivery using nanoparticles. Appl. Mater. Today 2022, 29, 101631 10.1016/j.apmt.2022.101631.

[ref17] Vecchi C. F.; Cesar G. B.; Souza P. R. D.; Caetano W.; Bruschi M. L. Mucoadhesive polymeric films comprising polyvinyl alcohol, polyvinylpyrrolidone, and poloxamer 407 for pharmaceutical applications. Pharm. Dev. Technol. 2021, 26 (2), 138–149. 10.1080/10837450.2020.1849283. 33183099

[ref18] Porfiryeva N. N.; Semina I. I.; Salakhov I. A.; Moustafine R. I.; Khutoryanskiy V. V. Mucoadhesive and mucus-penetrating interpolyelectrolyte complexes for nose-to-brain drug delivery. Nanomed. Nanotechnol. Biol. Med. 2021, 37, 102432 10.1016/j.nano.2021.102432. 34186258

[ref19] Xu Q.; Ensign L. M.; Boylan N. J.; Schön A.; Gong X.; Yang J.-C.; Lamb N. W.; Cai S.; Yu T.; Freire E.; Hanes J. Impact of Surface Polyethylene Glycol (PEG) Density on Biodegradable Nanoparticle Transport in Mucus ex Vivo and Distribution in Vivo. ACS Nano 2015, 9 (9), 9217–9227. 10.1021/acsnano.5b03876. 26301576 PMC4890729

[ref20] Conte G.; Costabile G.; Baldassi D.; Rondelli V.; Bassi R.; Colombo D.; Linardos G.; Fiscarelli E. V.; Sorrentino R.; Miro A.; Quaglia F.; Brocca P.; d’Angelo I.; Merkel O. M.; Ungaro F. Hybrid Lipid/Polymer Nanoparticles to Tackle the Cystic Fibrosis Mucus Barrier in siRNA Delivery to the Lungs: Does PEGylation Make the Difference?. ACS Appl. Mater. Interfaces 2022, 14 (6), 7565–7578. 10.1021/acsami.1c14975. 35107987 PMC8855343

[ref21] Guo Y.; Ma Y.; Chen X.; Li M.; Ma X.; Cheng G.; Xue C.; Zuo Y. Y.; Sun B. Mucus Penetration of Surface-Engineered Nanoparticles in Various pH Microenvironments. ACS Nano 2023, 17 (3), 2813–2828. 10.1021/acsnano.2c11147. 36719858

[ref22] Netsomboon K.; Bernkop-Schnürch A. Mucoadhesive vs. mucopenetrating particulate drug delivery. Eur. J. Pharm. Biopharm. 2016, 98, 76–89. 10.1016/j.ejpb.2015.11.003. 26598207

[ref23] Castillo Henríquez L.; Bahloul B.; Alhareth K.; Oyoun F.; Frejková M.; Kostka L.; Etrych T.; Kalshoven L.; Guillaume A.; Mignet N.; Corvis Y. Step-By-Step Standardization of the Bottom-Up Semi-Automated Nanocrystallization of Pharmaceuticals: A Quality By Design and Design of Experiments Joint Approach. Small 2024, 20 (25), e2306054 10.1002/smll.202306054. 38299478

[ref24] Bianchi M. B.; Zhang C.; Catlin E.; Sandri G.; Calderón M.; Larrañeta E.; Donnelly R. F.; Picchio M. L.; Paredes A. J. Bioadhesive eutectogels supporting drug nanocrystals for long-acting delivery to mucosal tissues. Materials Today Bio 2022, 17, 100471 10.1016/j.mtbio.2022.100471. PMC9636571 36345362

[ref25] Umerska A.; Gaucher C.; Oyarzun-Ampuero F.; Fries-Raeth I.; Colin F.; Villamizar-Sarmiento M. G.; Maincent P.; Sapin-Minet A. Polymeric Nanoparticles for Increasing Oral Bioavailability of Curcumin. Antioxidants 2018, 7 (4), 46 10.3390/antiox7040046. 29587350 PMC5946112

[ref26] Wright L.; Barnes T. J.; Joyce P.; Prestidge C. A. Optimisation of a High-Throughput Model for Mucus Permeation and Nanoparticle Discrimination Using Biosimilar Mucus. Pharmaceutics 2022, 14 (12), 2659 10.3390/pharmaceutics14122659. 36559151 PMC9782027

[ref27] Sanchez-Guzman D.; Boland S.; Brookes O.; Mc Cord C.; Lai Kuen R.; Sirri V.; Baeza Squiban A.; Devineau S. Long-term evolution of the epithelial cell secretome in preclinical 3D models of the human bronchial epithelium. Sci. Rep. 2021, 11 (1), 6621 10.1038/s41598-021-86037-0. 33758289 PMC7988136

[ref28] Smith A. M.; Nie S. Minimizing the Hydrodynamic Size of Quantum Dots with Multifunctional Multidentate Polymer Ligands. J. Am. Chem. Soc. 2008, 130 (34), 11278–11279. 10.1021/ja804306c. 18680294 PMC2703485

[ref29] Zhang K.; Xu Y.; Lu L.; Shi C.; Huang Y.; Mao Z.; Duan C.; Ren X. e.; Guo Y.; Huang C. Hydrodynamic cavitation: A feasible approach to intensify the emulsion cross-linking process for chitosan nanoparticle synthesis. Ultrason. Sonochem. 2021, 74, 105551 10.1016/j.ultsonch.2021.105551. 33894557 PMC8091060

[ref30] Mitchell M. J.; Billingsley M. M.; Haley R. M.; Wechsler M. E.; Peppas N. A.; Langer R. Engineering precision nanoparticles for drug delivery. Nat. Rev. Drug Discovery 2021, 20 (2), 101–124. 10.1038/s41573-020-0090-8. 33277608 PMC7717100

[ref31] Zong R.; Ruan H.; Zhu W.; Zhang P.; Feng Z.; Liu C.; Fan S.; Liang H.; Li J. Curcumin nanocrystals with tunable surface zeta potential: Preparation, characterization and antibacterial study. J. Drug Delivery Sci. Technol. 2022, 76, 103771 10.1016/j.jddst.2022.103771.

[ref32] Rask M. B.; Knopp M. M.; Olesen N. E.; Holm R.; Rades T. Comparison of two DSC-based methods to predict drug-polymer solubility. Int. J. Pharm. 2018, 540 (1–2), 98–105. 10.1016/j.ijpharm.2018.02.002. 29425764

[ref33] Kaewnopparat N.; Kaewnopparat S.; Jangwang A.; Maneenaun D.; Chuchome T.; Panichayupakaranant P. Increased Solubility, Dissolution and Physicochemical Studies of Curcumin- Polyvinylpyrrolidone K-30 Solid Dispersions. World Acad. Sci. Eng. Technol. 2009, 55, 229–234.

[ref34] Kowalczuk A.; Trzcinska R.; Trzebicka B.; Müller A. H.; Dworak A.; Tsvetanov C. B. Loading of polymer nanocarriers: Factors, mechanisms and applications. Prog. Polym. Sci. 2014, 39 (1), 43–86. 10.1016/j.progpolymsci.2013.10.004.

[ref35] Della Rocca J.; Liu D.; Lin W. Are high drug loading nanoparticles the next step forward for chemotherapy?. Nanomedicine 2012, 7 (3), 303–305. 10.2217/nnm.11.191. 22385191 PMC3777216

[ref36] Talevi A.; Bellera C. L. Biopharmaceutics Drug Disposition Classification System. In The ADME Encyclopedia: A Comprehensive Guide on Biopharmacy and Pharmacokinetics; Talevi A., Ed.; Springer International Publishing: Cham, 2022; pp 185–189.

[ref37] Lakshani N.; Wijerathne H. S.; Sandaruwan C.; Kottegoda N.; Karunarathne V. Release Kinetic Models and Release Mechanisms of Controlled-Release and Slow-Release Fertilizers. ACS Agric. Sci. Technol. 2023, 3 (11), 939–956. 10.1021/acsagscitech.3c00152.

[ref38] Dokoumetzidis A.; Macheras P. A century of dissolution research: from Noyes and Whitney to the biopharmaceutics classification system. Int. J. Pharm. 2006, 321 (1–2), 1–11. 10.1016/j.ijpharm.2006.07.011. 16920290

[ref39] Bilardo R.; Traldi F.; Vdovchenko A.; Resmini M. Influence of surface chemistry and morphology of nanoparticles on protein corona formation. Wiley Interdiscip. Rev. Nanomed. Nanobiotechnol. 2022, 14 (4), e1788 10.1002/wnan.1788. 35257495 PMC9539658

[ref40] Nance E.; Pun S. H.; Saigal R.; Sellers D. L. Drug delivery to the central nervous system. Nat. Rev. Mater. 2022, 7 (4), 314–331. 10.1038/s41578-021-00394-w. 38464996 PMC10923597

[ref41] Zhuang X.; Xiang X.; Grizzle W.; Sun D.; Zhang S.; Axtell R. C.; Ju S.; Mu J.; Zhang L.; Steinman L.; Miller D.; Zhang H. G. Treatment of brain inflammatory diseases by delivering exosome encapsulated anti-inflammatory drugs from the nasal region to the brain. Mol. Ther. 2011, 19 (10), 1769–1779. 10.1038/mt.2011.164. 21915101 PMC3188748

[ref42] Lee W.-H.; Loo C.-Y.; Young P. M.; Rohanizadeh R.; Traini D. Curcumin Nanoparticles Attenuate Production of Pro-inflammatory Markers in Lipopolysaccharide-Induced Macrophages. Pharm. Res. 2016, 33 (2), 315–327. 10.1007/s11095-015-1789-9. 26350106

[ref43] Derosa G.; Maffioli P.; Simental-Mendía L. E.; Bo S.; Sahebkar A. Effect of curcumin on circulating interleukin-6 concentrations: A systematic review and meta-analysis of randomized controlled trials. Pharmacol. Res. 2016, 111, 394–404. 10.1016/j.phrs.2016.07.004. 27392742

[ref44] Sibinovska N.; Žakelj S.; Trontelj J.; Kristan K. Applicability of RPMI 2650 and Calu-3 Cell Models for Evaluation of Nasal Formulations. Pharmaceutics 2022, 14 (2), 369 10.3390/pharmaceutics14020369. 35214101 PMC8877043

[ref45] Inoue D.; Furubayashi T.; Tanaka A.; Sakane T.; Sugano K. Quantitative estimation of drug permeation through nasal mucosa using in vitro membrane permeability across Calu-3 cell layers for predicting in vivo bioavailability after intranasal administration to rats. Eur. J. Pharm. Biopharm. 2020, 149, 145–153. 10.1016/j.ejpb.2020.02.004. 32057906

[ref46] He Y.; Liang Y.; Mak J. C. W.; Liao Y.; Li T.; Yan R.; Li H. F.; Zheng Y. Size effect of curcumin nanocrystals on dissolution, airway mucosa penetration, lung tissue distribution and absorption by pulmonary delivery. Colloids Surf., B 2020, 186, 110703 10.1016/j.colsurfb.2019.110703. 31835185

