# Supplementary material for: Unveiling the Potential of Surface Polymerized Drug Nanocrystals in Targeted Delivery
Source: ACS Appl Mater Interfaces. 2024 Aug 28;16(36):47124–36. doi: 10.1021/acsami.4c07669 (PMC11403545; doi:10.1021/acsami.4c07669)
Supplement: Supplementary file 1 — am4c07669_si_001.pdf [file am4c07669_si_001.pdf]

Supporting information

Unveiling the Potential of Surface Polymerized Drug Nanocrystals in Targeted Delivery

Jakes Udabe[1], Sergio Martin-Saldaña[1], Yushi Tao[2], Matías Picchio[1], Ana Beloqui[1,3], Alejandro J. Paredes[2*], Marcelo Calderón[1,3*]

[1] POLYMAT, Applied Chemistry Department, Faculty of Chemistry, University of the Basque Country UPV/EHU, Paseo Manuel de Lardizabal 3, 20018 Donostia - San Sebastián, España.

[2] School of Pharmacy, Queen's University Belfast, 97 Lisburn Road, Belfast, Northern Ireland, BT9 7BL. United Kingdom.

[3] IKERBASQUE, Basque Foundation for Science, Plaza Euskadi 5, 48009, Bilbao, España.

*Corresponding authors:

Marcelo Calderón (e-mail: marcelo.calderonc@ehu.eus)

Alejandro J. Paredes (e-mail: A.Paredes@qub.ac.uk)

INDEX

Table S1. Regression analysis of cumulative drug release curves for Raw Curcumin using various theoretical models. 4

Table S2. Regression analysis of cumulative drug release curves for Initial NCs using various theoretical models. 4

Table S3. Regression analysis of cumulative drug release curves for Control NCs using various theoretical models. 4

Table S4. Regression analysis of cumulative drug release curves for HC NCs using various theoretical models. 5

Table S5. Regression analysis of cumulative drug release curves for LC NCs using various theoretical models. 5

Table S6. Rate constants comparison (k values) of the Baker and Lonsdale model. 6

Figure S1. (A) NCs stability in MilliQ water at room temperature, (B) Hydrodynamic size before and after freeze-drying. 6

Figure S2. Stability of the NCs in 10 mM PBS pH = 7.4 at 4 ºC 7

Figure S3. Stability of the NCs in 10 mM PBS pH = 7.4 at RT 7

Figure S4. Stability of the NCs in 10 mM PBS pH = 7.4 and 37 ºC 8

Figure S5. Control ^{1}H-NMR of Raw curcumin, polaxamer dyacrilate and polaxamer 8

Figure S6. ^{1}H-NMR spectra of the different formulations of initial NCs and Raw curcumin 9

Figure S7.^{13}C-NMR spectra of the different formulations of Control NCs and Raw curcumin........10

Figure S8. ^{1}H-NMR spectra of the different formulations of control NCs and Raw curcumin........10

Figure S9. ^{1}H-NMR spectra of the different formulations of higly crosslinked surface PEG NCs (HCS NCs) and Raw curcumin........11

Figure S10. ^{1}H-NMR spectra of the different formulations of low crosslinked surface PEG NCs (LC NCs) and Raw curcumin........11

Figure S11. FTIR spectra of the different NCs variations. 12

Figure S12. DSC curves of the different NCs variations. 13

Figure S13. Control DSC curves of the different materials 14

Figure S14. CUR release kinetics in 10 mM PBS 7.4 and 10 mM PBS 7.4 with 1% ascorbic acid. 15

Figure S15. Effect on the (A) TNFα, (B) IL-1β, and (C) IL-6 release from THP-1, derived macrophages 15

Figure S16. Lucifer yellow assay 16

Figure S17. CUR based formulations uptake after 4 h exposure on HMC3. Micrographs of HMC3 cells after 4 h incubation with complete media (A), raw CUR (B), Initial NCs (C), Control NC (D), HCS NCs (E) and LCS NCs (F) 16

Figure S18. CUR based formulations uptake after 24 h exposure on HMC3. Micrographs of HMC3 cells after 24 h incubation with complete media (A), raw CUR (B), Initial NCs (C), Control NC (D), HCS NCs (E) and LCS NCs (F)..17

Table S1. Regression analysis of cumulative drug release curves for raw curcumin using various theoretical models.

Model/equation	Raw Curcumin
Zero Order	0.965
First Order	0.974
Hixson and Crowell	0.941
Baker and Lonsdale	**0.997**
Higuchi	0.878

Table S2. Regression analysis of cumulative drug release curves for Initial NCs using various theoretical models.

Model/equation	100_0	75_25	50_50	25_75	0_100
Zero Order	0.985	0.840	0.867	0.885	0.852
First Order	0.976	0.892	0.967	0.939	**0.966**
Hixson and Crowell	0.894	0.824	0.936	0.872	0.80
Baker and Lonsdale	**0.995**	**0.921**	**0.982**	**0.936**	0.899
Higuchi	0.823	0.759	0.894	0.824	0.774

Table S3. Regression analysis of cumulative drug release curves for Control NCs using various theoretical models.

Model/equation	100_0	75_25	50_50	25_75	0_100
Zero Order	0.984	0.912	0.874	0.931	0.976
First Order	0.920	0.918	0.884	0.922	0.943
Hixson and Crowell	0.996	0.874	0.868	0.862	0.887
Baker and Lonsdale	**0.967**	**0.948**	**0.913**	**0.959**	**0.963**
Higuchi	0.899	0.835	0.836	0.815	0.847

Table S4. Regression analysis of cumulative drug release curves for HC NCs using various theoretical models.

**Model/equation**	**100_0**	**75_25**	**50_50**	**25_75**	**0_100**
Zero Order	0.978	0.870	0.978	0.907	0.893
First Order	0.933	0.878	0.959	0.878	0.825
Hixson and Crowell	0.880	0.826	0.909	0.802	0.745
Baker and Lonsdale	**0.961**	**0.984**	**0.988**	**0.914**	**0.907**
Higuchi	0.806	0.756	0.837	0.722	0.675

Table S5. Regression analysis of cumulative drug release curves for LC NCs using various theoretical models.

**Model/equation**	**100_0**	**75_25**	**50_50**	**25_75**	**0_100**
Zero Order	0.941	0.937	0.927	0.955	0.952
First Order	0.971	0.975	0.967	0.974	0.972
Hixson and Crowell	0.914	0.933	0.923	0.918	0.915
Baker and Lonsdale	**0.991**	**0.995**	**0.990**	**0.994**	**0.993**
Higuchi	0.839	0.864	0.855	0.842	0.842

Table S6. Rate constants comparison (k values) of the Baker and Lonsdale model.

Raw NCs	-0.03598

	100_0	75_25	50_50	25_75	0_100
Initial NCs	-0.04422	-0.04018	-0.04048	-0.04208	-0.04243
Control NCs	-0.03942	-0.03822	-0.04079	-0.03917	-0.03831
HC NCs	-0.03937	-0.04030	-0.03936	-0.03881	-0.03764
LC NCs	-0.03650	-0.03659	-0.03778	-0.03700	-0.03907

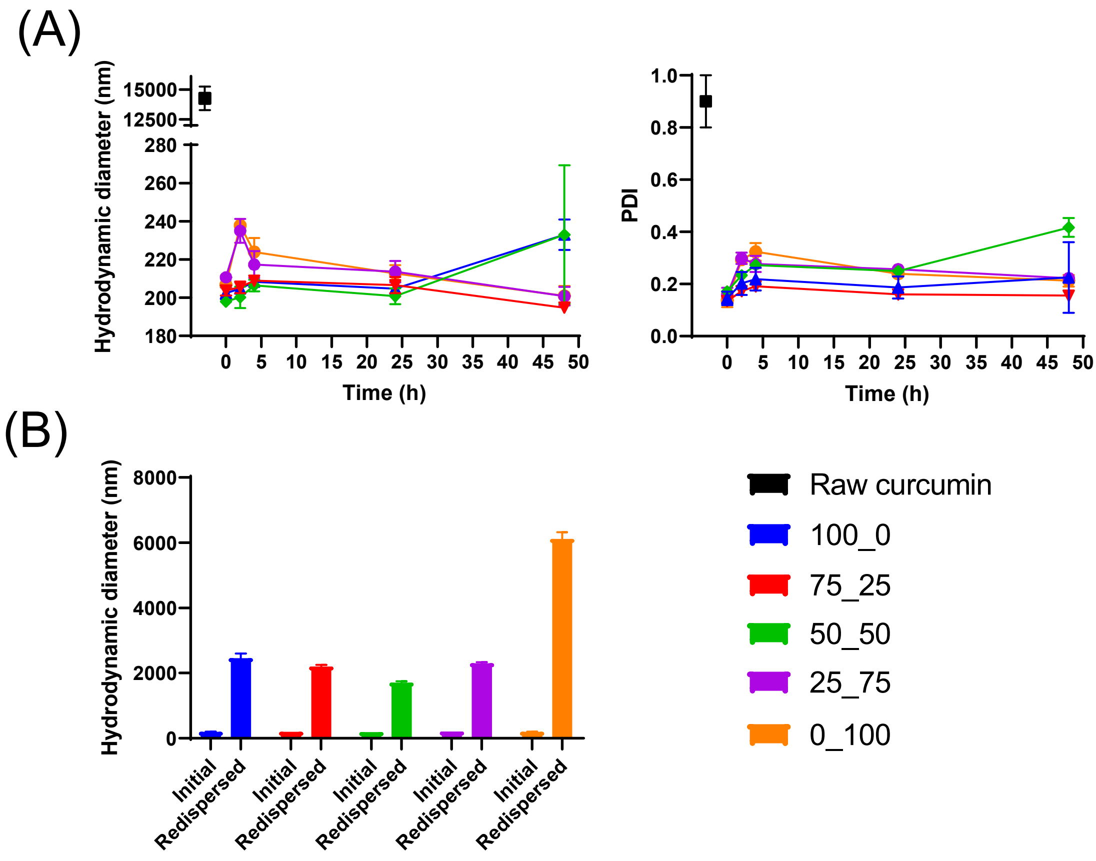

Figure S1. (A) NCs stability in terms of size and PDI during dialysis measured in MilliQ water at room temperature (RT) at 1 mg/mL (B) Hydrodynamic size before and after freeze-drying. All the data were measured by intensity distribution by triplicate (n=3), and all the data are presented as mean ± SD.

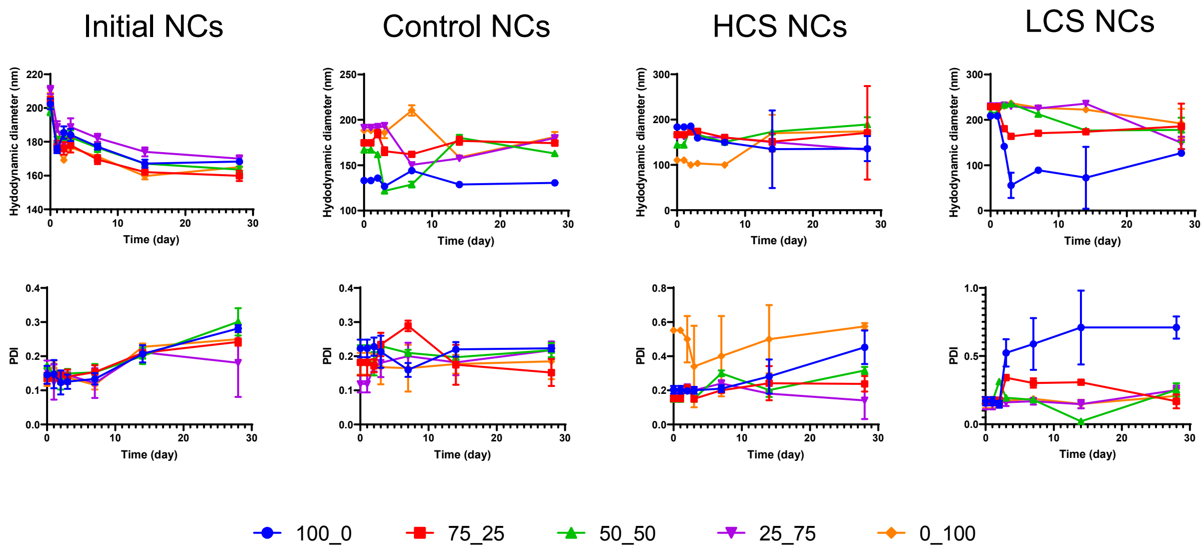

Figure S2. Stability of the NCs measured by DLS in 10 mM PBS pH = 7.4 at 4 ºC. All the data were measured by intensity distribution by triplicate (n=3), and all the data are presented as mean ± SD.

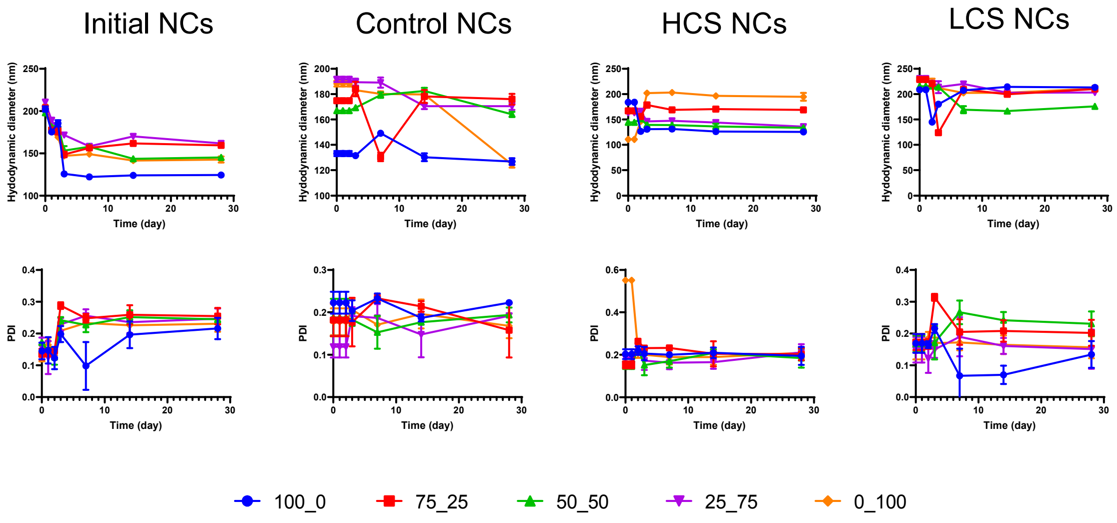

Figure S3. Stability of the NCs measured by DLS in 10 mM PBS pH = 7.4 at RT. All the data were measured by intensity distribution by triplicate (n=3), and all the data are presented as mean ± SD.

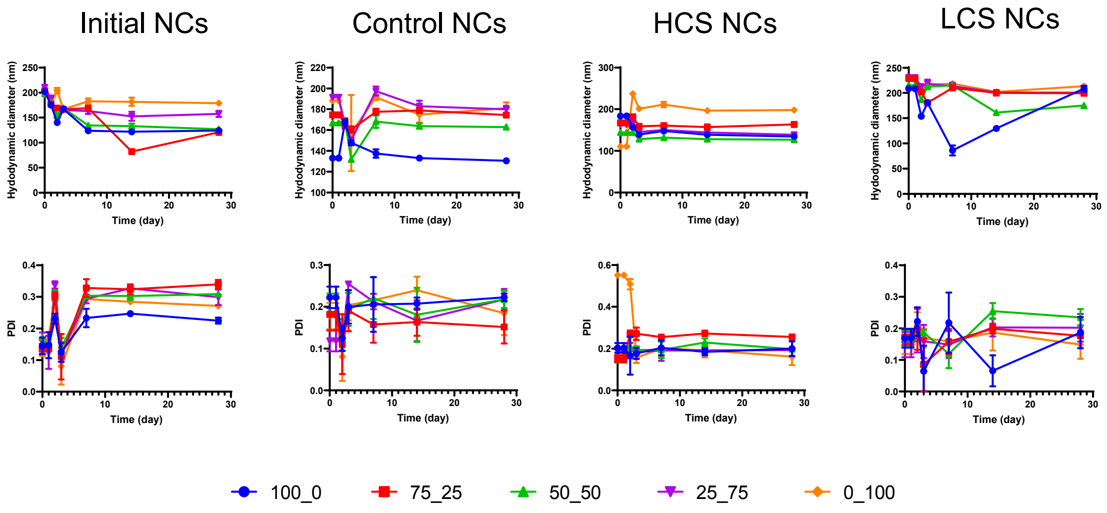

Figure S4. Stability of the NCs measured by DLS in 10 mM PBS pH = 7.4 and 37 ºC. All the data were measured by intensity distribution by triplicate (n=3), and all the data are presented as mean ± SD.

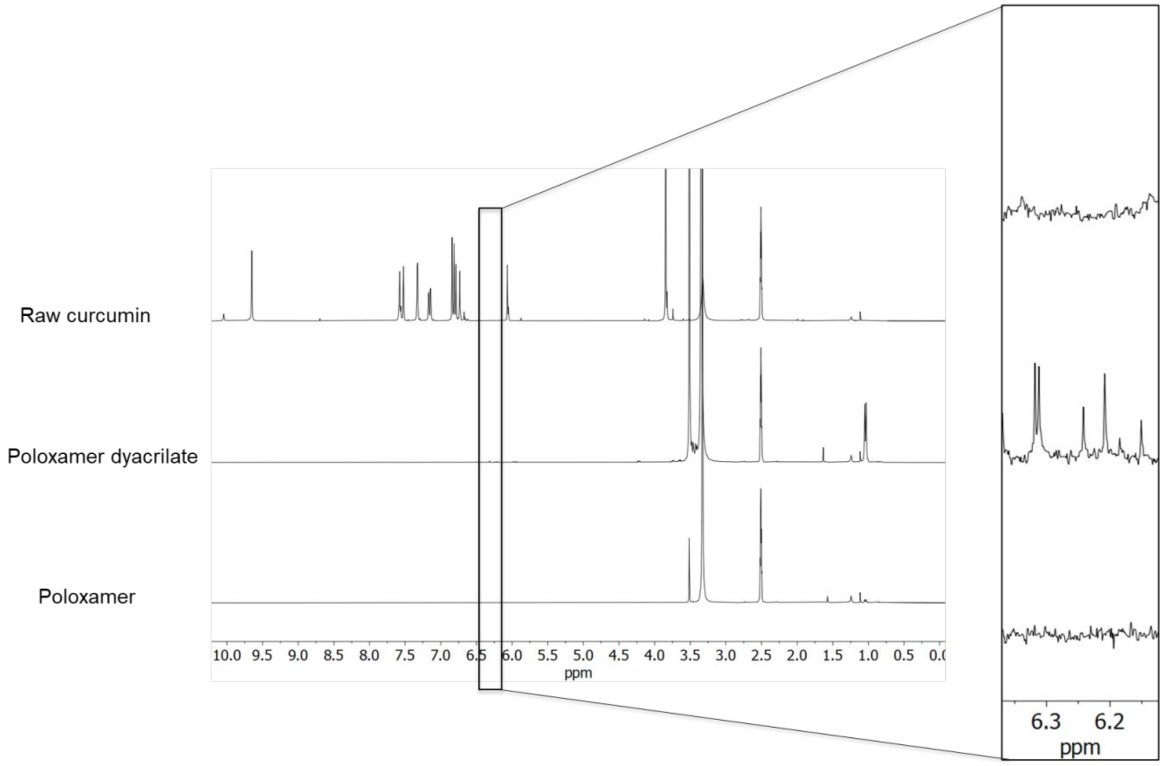

Figure S5. Control H-NMR of Raw curcumin, polaxamer dyacrilate and polaxamer measured in Bruker AVANCE 300 by dissolving 20 mg of the NCs in 600 µL DMSO-d9.

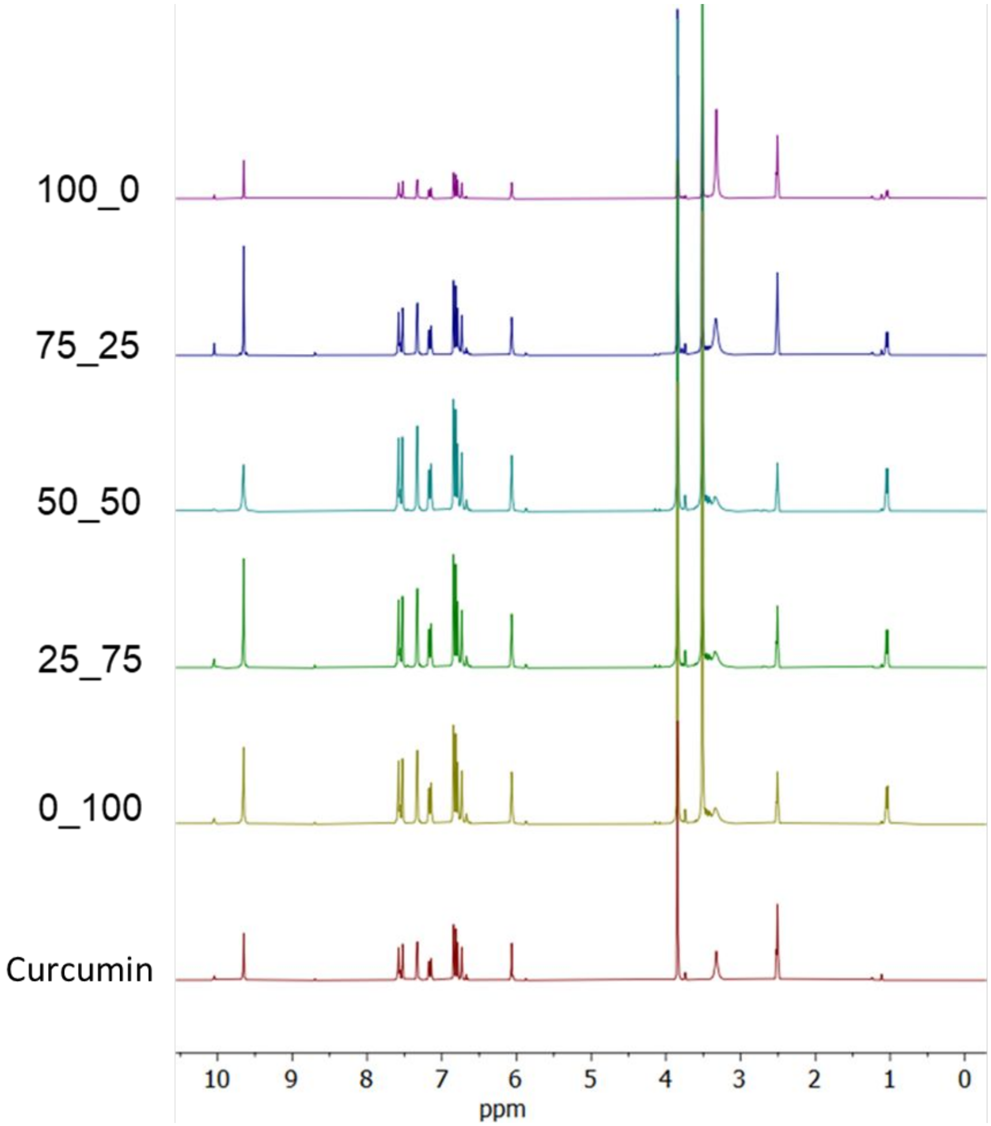

Figure S6. ^{1}H-NMR spectra of the different formulations of initial NCs and Raw curcumin, measured in Bruker AVANCE 300 by dissolving 20 mg of the NCs in 600 µL DMSO-d9.

As depicted in Figure S5, the H-1 signal exhibited a singlet with a chemical shift of δ 5.80 ppm. The H-3 signal appeared as a doublet at δ 6.47 ppm with a coupling constant of 15.76 Hz. In contrast, the H-4 signal experienced a shift to δ 7.590 ppm

with the same coupling constant. Ultimately, the signals corresponding to the aromatic hydrogens displayed shifts from 6.90 to 7.15 ppm. Therefore, it can be deduced that the curcumin maintained its chemical structure.

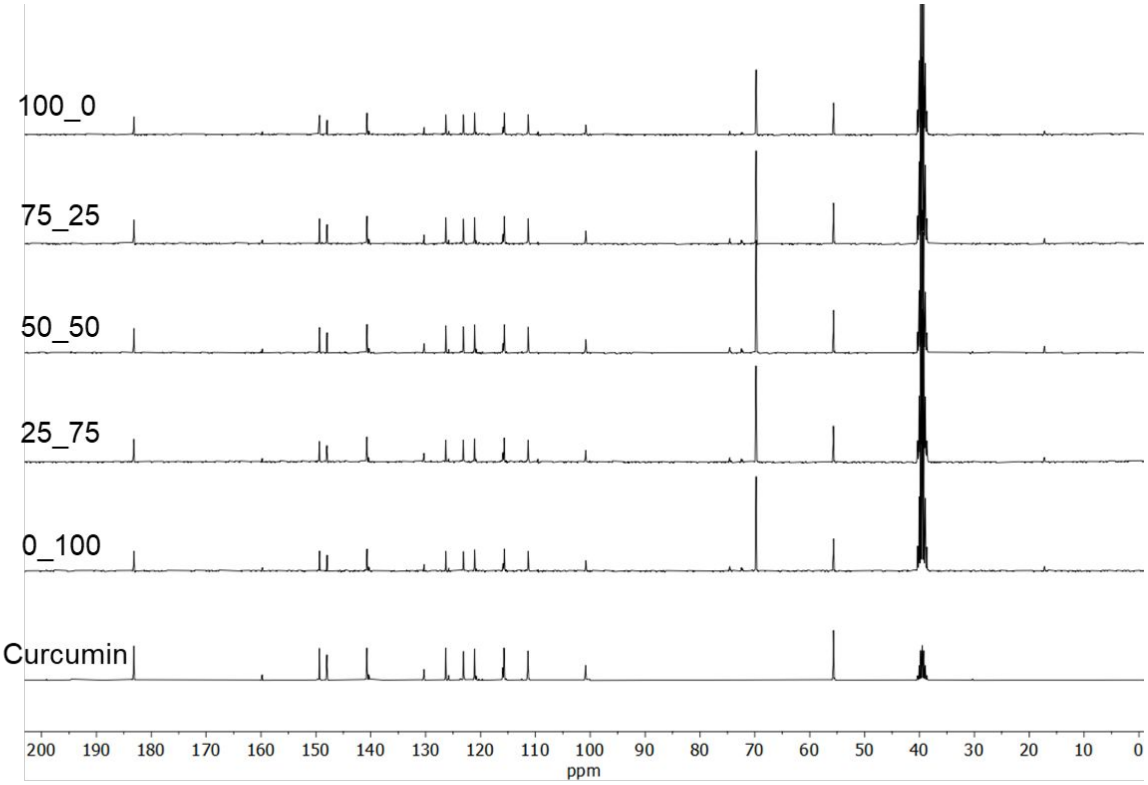

Figure S7. ^{13}C-NMR spectra of the different formulations of Control NCs and Raw curcumin, measured in Bruker AVANCE 300 by dissolving 20 mg of the NCs in 600 µL DMSO-d9.

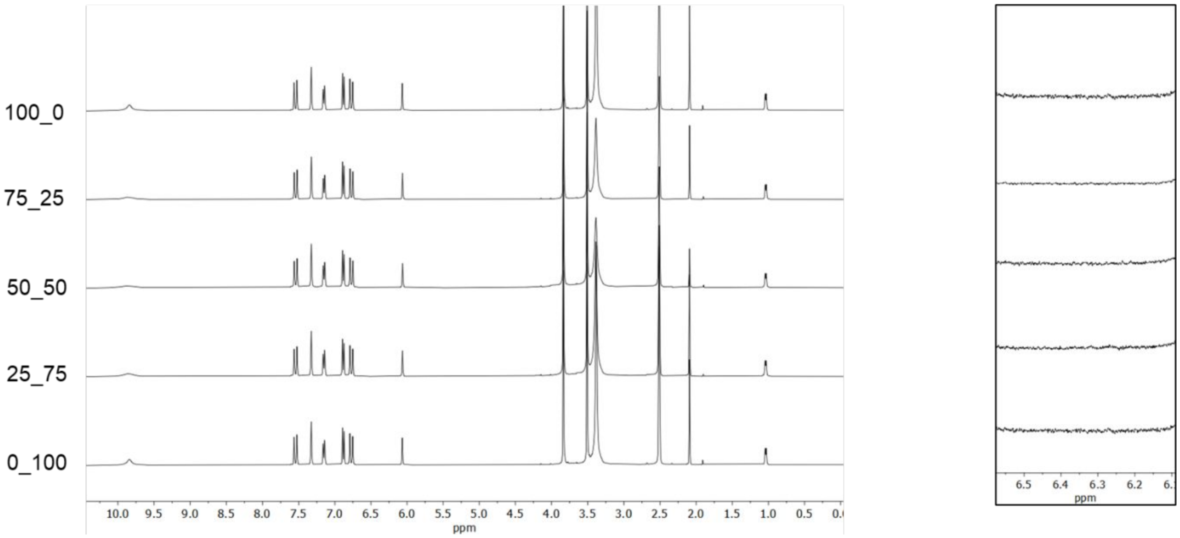

Figure S8. ^{1}H-NMR spectra of the different formulations of control NCs and Raw curcumin, measured in Bruker AVANCE 300 by dissolving 20 mg of the NCs in 600 µL DMSO-d9. On the right side of the figure, the zoomed-in 1H-NMR spectrum is located, ranging between 6.1 and 6.6 ppm in the region corresponding to acrylic groups.

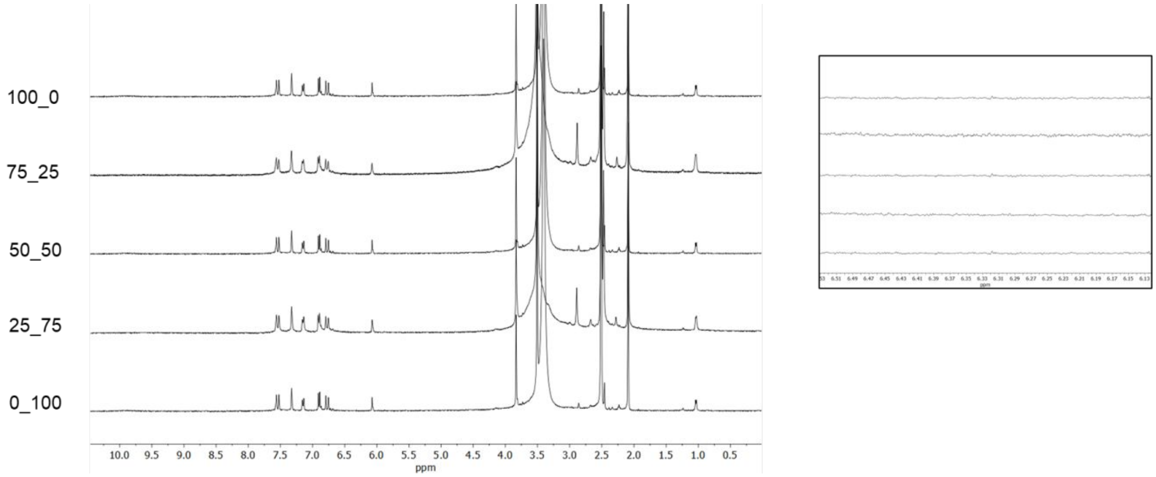

Figure S9. ^{1}H-NMR spectra of the different formulations of higly crosslinked surface PEG NCs (HCS NCs) and Raw curcumin, measured in Bruker AVANCE 300 by dissolving 20 mg of the NCs in 600 µL DMSO-d9. On the right side of the figure, the zoomed-in 1H-NMR spectrum is located, ranging between 6.1 and 6.6 ppm in the region corresponding to acrylic groups.

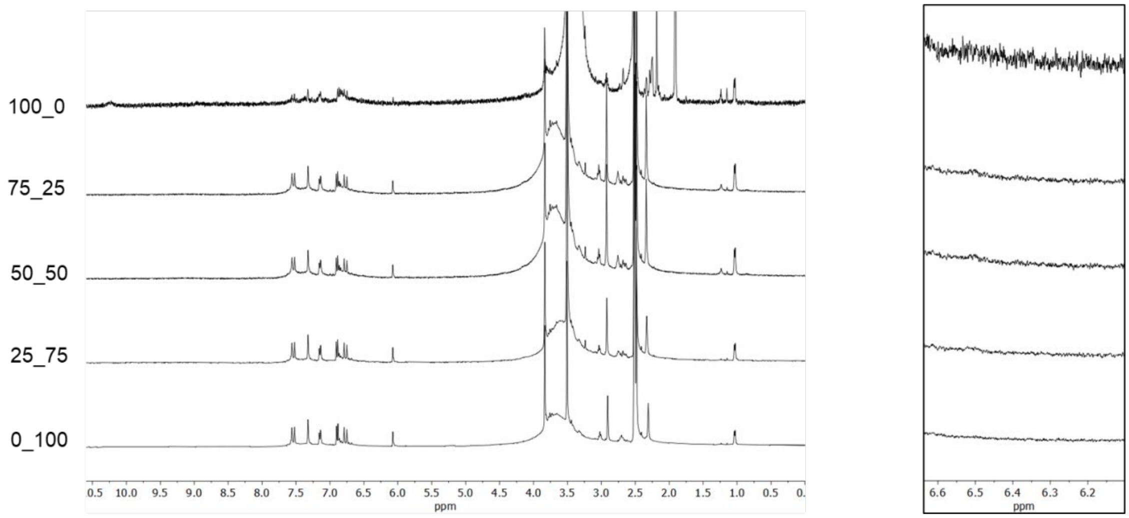

Figure S10. ^{1}H-NMR spectra of the different formulations of low crosslinked surface PEG NCs (LC NCs) and Raw curcumin, measured in Bruker AVANCE 300 by dissolving 20 mg of the NCs in 600 µL DMSO-d9. On the right side of the figure, the

zoomed-in 1H-NMR spectrum is located, ranging between 6.1 and 6.6 ppm in the region corresponding to acrylic groups.

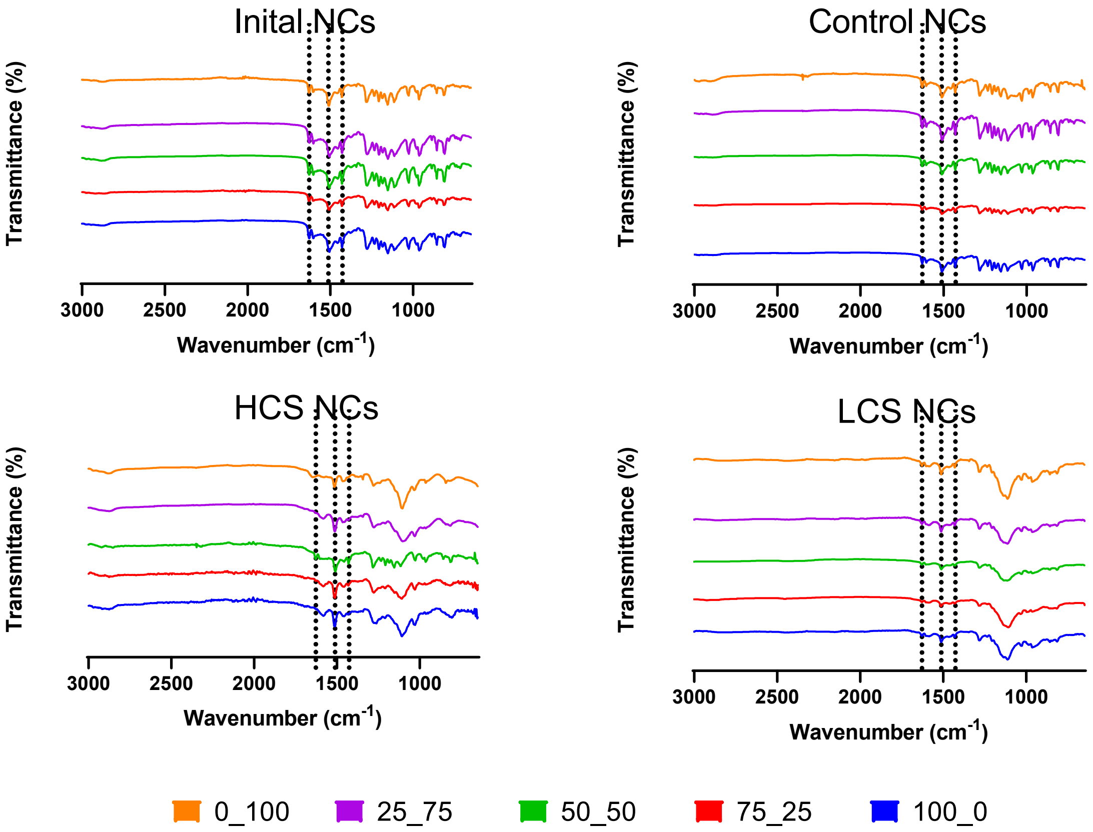

Figure S11. FTIR spectra of the different NCs variations.

FTIR spectra illustrate characteristic signals at 1628 cm^{-1} primarily attributed to the overlapping stretching vibrations characteristic of alkenes (C=C) and carbonyl (C=O) functionalities. The infrared spectrum of the curcumin reveals aromatic C=C stretching vibrations at 1427 cm^{-1}, and a high-intensity band at 1512 cm^{-1}, associated with mixed vibrations encompassing stretching carbonyl bond stretching (ν (C=O)), in-plane bending vibrations of aliphatic (δ CC-C, δ CC=O), and in-plane bending vibrations of aromatic (δ CC-H) configurations of keto and enol forms. Additionally, stretching vibrations of aromatic (ν CC) bonds of both keto and enolic forms of curcumin are evident. Therefore, the structure of curcumin can be determined.

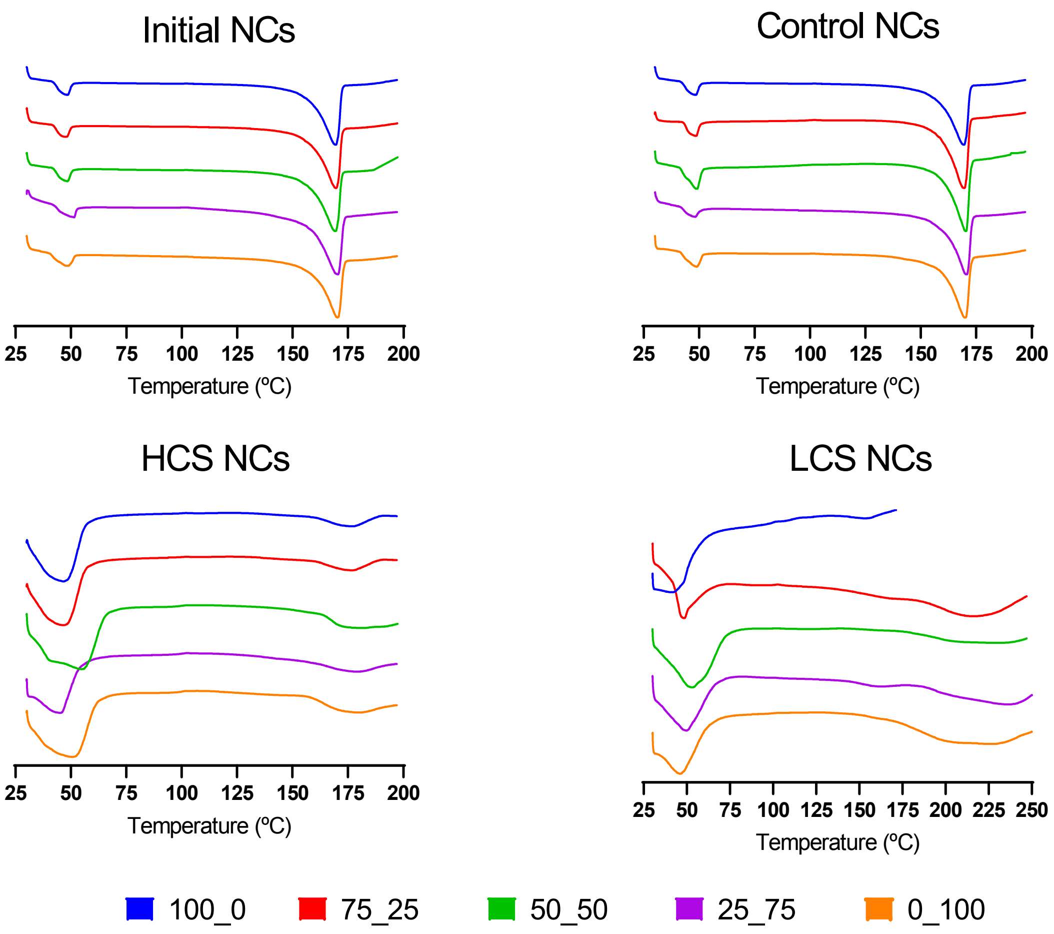

Figure S12. DSC curves of the different NCs variations. DSC was performed using a Q100 differential scanning calorimeter system (TA Instruments, New Castle, DE, USA). The samples (5-10 mg) were placed into standard aluminum pans and heated in a nitrogen atmosphere at a stepping rate of 10 °C/min over a temperature range of 0 °C to 200 °C with an empty pan as reference.

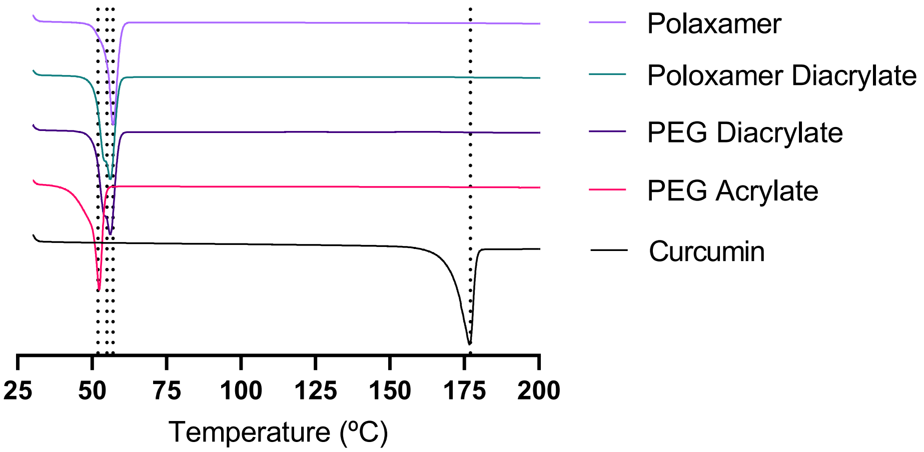

Figure S13. Control DSC curves of the different materials. DSC was performed using a Q100 differential scanning calorimeter system (TA Instruments, New Castle, DE, USA). The samples (5-10 mg) were placed into standard aluminium pans and heated in a nitrogen atmosphere at a stepping rate of 10 °C/min over a temperature range of 0 °C to 200 °C with an empty pan as reference.

Using differential scanning calorimetry (DSC) we could discern the characteristic bands of the polymers. After conducting control experiments with only the polymers, similar trends were observed in the curves. Furthermore, overlapping of the bands is evident when there are mixtures of different polymers. Therefore, we can confirm that the nanocrystals contain polymeric structures.

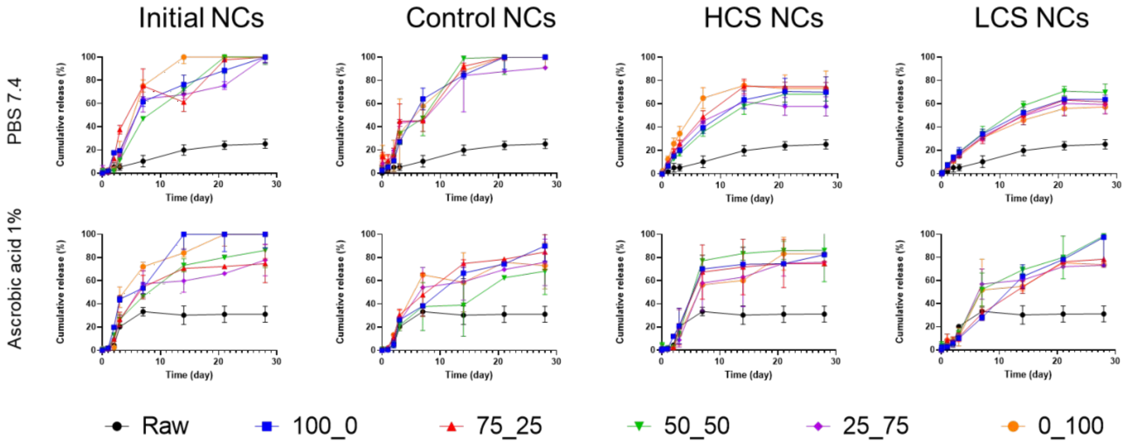

Figure S14. CUR release kinetics in 10 mM PBS 7.4 and 10 mM PBS 7.4 with 1% ascorbic acid.

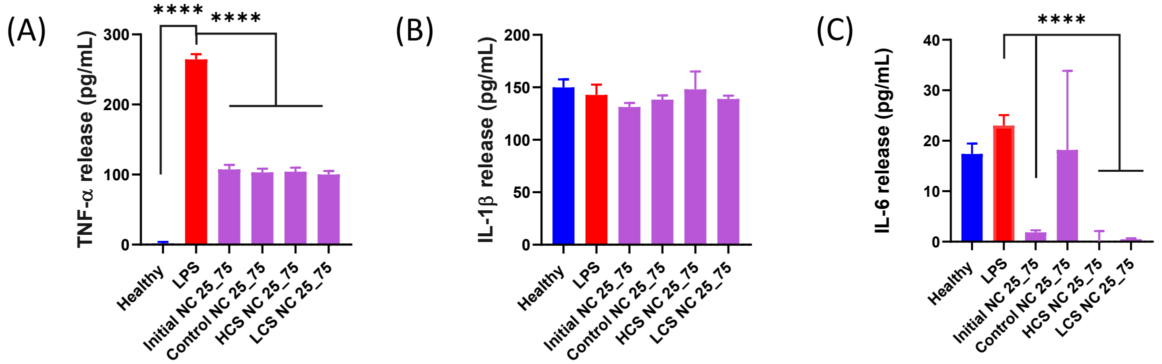

Figure S15. Effect on the (A) TNFα, (B) IL-1β, and (C) IL-6 release from THP-1, derived macrophages measured as the concentration in the supernatant after the treatment. Data obtained from N >3. Mean ± standard deviation (SD), single ANOVA with Tukey multiple comparisons test was used *$p < 0.05$; **$p < 0.01$. ***$p < 0.005$; ****$p < 0.001$ compared with control group.

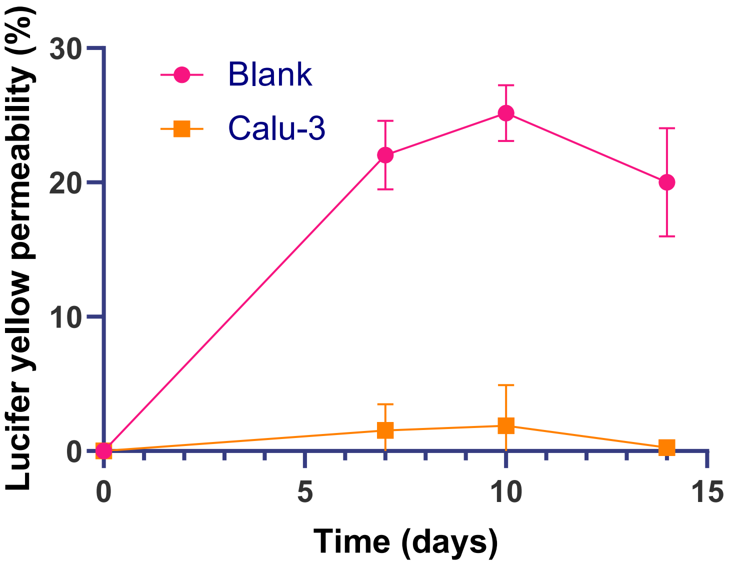

Figure S16. Lucifer yellow assay. 1 µg/mL Lucifer yellow solution was added to the upper part of the trasnwell and 100 µL of the acceptor chamber were withdrawn at selected timepoints and compared to the fluorescence in transwell without cells seeded. Data obtained from n >3. Mean ± standard deviation (SD).

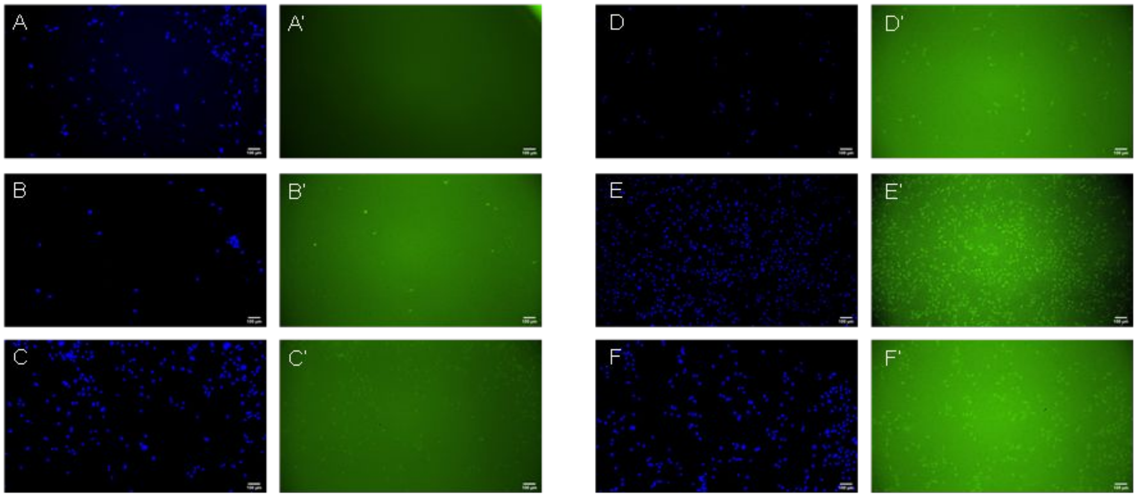

Figure S17. CUR based formulations uptake after 4 h exposure on HMC3. Micrographs of HMC3 cells after 4 h incubation with complete media (A), raw CUR (B), Initial NCs (C), Control NC (D), HCS NCs (E) and LCS NCs (F). Three independent experiments (n = 3) were performed, and representative images are shown here for nuclei (A-F) or FITC channel (A'-F'). Scale bars: 100 µm.

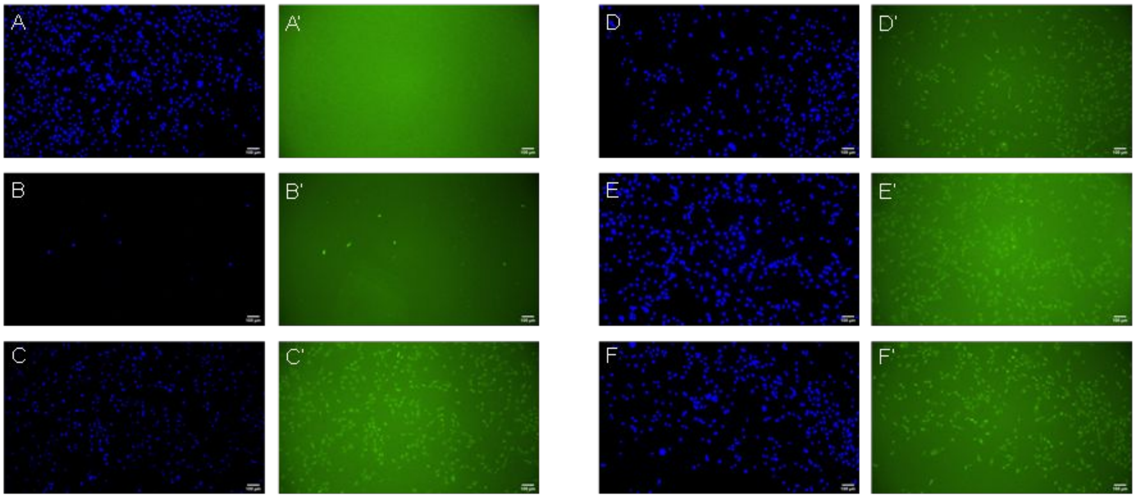

Figure S18. CUR based formulations uptake after 24 h exposure on HMC3. Micrographs of HMC3 cells after 24 h incubation with complete media (A), raw CUR (B), Initial NCs (C), Control NC (D), HCS NCs (E) and LCS NCs (F). Three independent experiments (n = 3) were performed, and representative images are shown here for nuclei (A-F) or FITC channel (A'-F'). Scale bars: 100 µm.
